# Ionic Liquids in Pharmaceuticals: A Scoping Review of Formulation Strategies

**DOI:** 10.1021/acsomega.5c08558

**Published:** 2026-01-01

**Authors:** Igor Luiz Gonçalves Pereira, Ana Luiza Ziulkoski, Karine Modolon Zepon, Luiz Alberto Kanis, Henri Stephan Schrekker

**Affiliations:** † Institute of Chemistry, Laboratory of Technological Processes and Catalysis, 28124 Universidade Federal do Rio Grande do Sul, Av. Bento Gonçalves, 9500, 91501-970 Porto Alegre, RS, Brazil; ‡ Institute of Health Sciences, Laboratory of Cytotoxicity, Universidade Feevale, RS-239, 2755, 93510-250 Novo Hamburgo, RS, Brazil; § Universidade do Sul de Santa Catarina, Ciências da Saúde, Av. José Acácio Moreira, 787, 88704-900 Tubarão, SC, Brazil; ∥ Airela Indústria Farmacêutica Ltda., SC-390, 500, 88720-000 Pedras Grandes, SC, Brazil

## Abstract

Ionic liquids (ILs) have rapidly emerged as multifunctional formulation tools capable of addressing long-standing challenges in drug delivery. This scoping review systematically assessed their pharmaceutical applications through a structured search of Scopus, PubMed, and Web of Science, yielding 1866 records, of which 91 experimental studies met the inclusion criteria. Across oral, injectable, topical, transdermal, nasal, and advanced delivery systems, ILs consistently improved the drug solubility, chemical stability, mucosal permeability, and overall biopharmaceutical performance. Choline- and imidazolium-based ILs were the most frequently investigated, demonstrating favorable safety profiles and significant pharmacokinetic benefits—including increases in maximum plasma concentration (*C*_max_), area under the concentration–time curve (AUC), and duration of action for poorly soluble small molecules, peptides, and biologics. Beyond classical excipient roles, ILs acted as structural and functional components in next-generation delivery platforms such as self-emulsifying systems, ionogels, microneedles, polymer–IL nanocomposites, and mucoadhesive films. Mechanistic evidence highlights their capacity to modulate molecular solvation, disrupt biological barriers, enable reversible tight-junction opening, and stabilize labile biomacromolecules. Collectively, these findings establish ILs as versatile and tunable design elements capable of overcoming key limitations in drug solubility and bioavailability. However, despite substantial preclinical progress, the clinical translation of IL-based formulations remains limited by regulatory uncertainty and the need for standardized toxicological and environmental safety evaluations.

## Graphical abstract

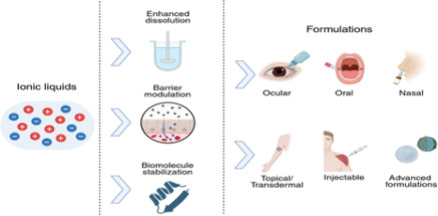

## 1 Introduction

Ionic liquids (ILs) are organic salts composed of asymmetric ion pairs, typically combining an organic cation with an inorganic or organic anion and exhibiting melting points of up to 100 °C.^1^ Their molecular diversity enables the rational design of systems with tailored physicochemical and biological properties,^2^ including solubility, stability,^3,4^ permeability, and biocompatibility.^5^ Because of their low volatility, structural tunability, and compatibility with both hydrophilic and hydrophobic environments, ILs have become increasingly relevant in pharmaceutical sciences, particularly in drug delivery and formulation design.^2,6^

Over the past decade, ILs have attracted growing attention for their dual functionality as both active pharmaceutical ingredients (APIs) and functional excipients capable of improving dissolution, permeability, and bioavailability. Choline-based systems, such as CAGE ([Cho]­[Ger] and [Cho]­[Gly])^7,8^ have demonstrated excellent biocompatibility and solubilization capacity, while imidazolium salts, including [C_4_MIm]­[BF_4_] and [C_6_MIm]­[Cl], serve as model frameworks to probe structure–property relationships in drug solubilization and intestinal transport.^9^ These representative ILs have inspired the development of more complex delivery platforms such as self-emulsifying carriers, ionogels, microneedles, and mucoadhesive films, broadening their pharmaceutical applicability.^10–12^

Despite numerous reviews addressing the biological activity,^13–15^ toxicity,^16,17^ and general biomedical uses^1,5,18–20^ of ILs, none have systematically connected ionic structure with formulation performance across distinct administration routes. Recent reviews have continued to discuss the growing pharmaceutical relevance of ILs, including toxicological challenges in IL-based drug delivery,^21,22^ functional ILs as pharmaceutical excipients,^23^ and API–IL pairing strategies to modulate solubility and dissolution.^24^ In parallel, recent works examined ILs in advanced formulation contexts, including ionogels and transdermal systems.^25–27^ However, these publications mostly treat ILs as isolated case studies or as physicochemical modifiers rather than integrating them across oral, parenteral, topical, and nasal routes of administration.

To address this gap, this review synthesizes data from 98 experimental studies retrieved from Scopus, PubMed, and Web of Science that define the current landscape of IL-based pharmaceutical formulations. The analysis focuses on how ILs address long-standing formulation challenges, such as poor aqueous solubility, low permeability, and chemical instability, while mapping toxicological, regulatory, and translational constraints. A graphical overview of their dual role across administration routes is provided in Figure 1. By integrating mechanistic, pharmacokinetic, and biocompatibility data, this review proposes a unified framework linking IL structure, formulation function, and biological performance with the aim of guiding the rational development of next-generation IL-enabled therapeutics.

**Figure 1:**
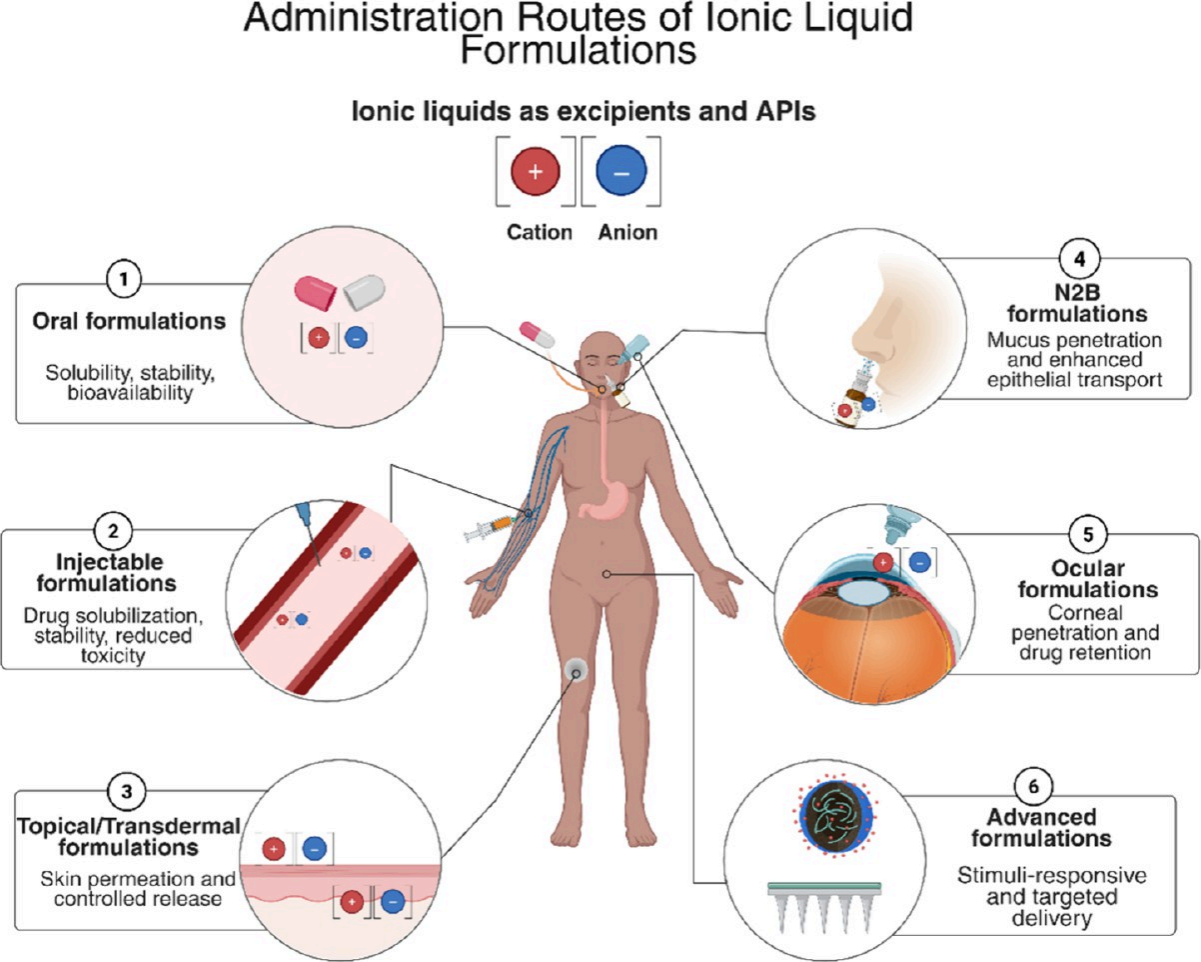
Schematic overview of ILs in pharmaceutical formulations highlighting their dual role as API-ILs and functional excipients. Representative administration routes include oral, injectable, topical/transdermal, ocular, intranasal and advanced systems, where ILs contribute to enhanced solubility, stability, permeability, and therapeutic performance. Created in BioRender. Pereira, I. (2026) https://BioRender.com/z53ci4k.

## 2 Search Strategy and Selection Criteria

A comprehensive literature search was conducted in December 2024 across three electronic databases: Scopus, PubMed, and the Web of Science. The search strategy combined terms related to ILs and their pharmaceutical or therapeutic applications, using the following query string: (“Ionic Liquids” OR “Biocompatible Ionic Liquids” OR “Functionalized Ionic Liquids”) AND (“Pharmaceutical Formulations” OR “Drug Formulations” OR “Pharmaceutical Applications” OR “Controlled Release Formulations”).

To ensure inclusion of studies focusing on intranasal and nose-to-brain (N2B) delivery—an emerging field within pharmaceutical ionic-liquid research—a targeted complementary search was conducted in October 2025 using the following query: (“Ionic Liquid*” OR “Ionogel*”) AND (“Intranasal” OR “Nasal Delivery” OR “Nose-to-Brain” OR “Olfactory Transport”) AND (“Drug Delivery” OR “Pharmaceutical” OR “Therapeutic”). This additional search resulted in 20 records. After removing duplicates and nonpharmaceutical studies, 7 articles met the inclusion criteria and were incorporated into the data set.

In total, 1873 records were initially identified across all databases. After eliminating 561 duplicates, 1312 unique articles were screened by a single reviewer using Rayyan QCRI,^28^ a web-based platform for systematic review management. Screening proceeded in two stages: first, titles and abstracts were assessed for relevance, resulting in the exclusion of 1067 articles. The remaining 245 articles underwent full-text review based on predefined eligibility criteria.

Studies were included if they described the application of ILs in pharmaceutical or therapeutic formulations, encompassing oral, injectable, topical, transdermal, and intranasal (N2B) systems, and if they reported in vitro and/or in vivo data on biocompatibility, efficacy, or pharmacokinetic performance. Exclusion criteria comprised review papers, non-English publications, studies unrelated to the pharmaceutical use of ILs, or those focused solely on physicochemical or nontherapeutic applications.

After full-text screening, 98 studies were included in the final data set—comprising 91 originally retrieved papers and seven N2B-related publications identified in the targeted 2025 search. Data extraction was performed using a standardized form that captured the publication year, chemical structure, and IL type, as well as formulation category, experimental model (in vitro and/or in vivo), and key outcomes related to solubility, stability, permeability, pharmacokinetics, efficacy, and safety.

To maintain methodological transparency and reproducibility, only studies meeting the predefined inclusion criteria were analyzed; consequently, nontargeted or peripheral publications not identified through the structured search were excluded, even when related to the broader IL formulation field.

## 3 Ionic Liquids for Pharmaceutical Formulation Design

### 3.1 Oral Delivery Systems

The oral route remains the most convenient and widely used administration pathway in pharmacotherapy, offering excellent patient compliance and flexibility in formulation design. However, many APIs suffer from poor aqueous solubility, low permeability, and chemical instability, which compromise their bioavailability and therapeutic efficacy. ILs have emerged as a modular platform to overcome these challenges by enhancing solubility in aqueous or lipid media, improving chemical stability, and facilitating intestinal permeability.

Owing to their amphiphilic nature, ILs can interact with both hydrophilic and lipophilic environments, enabling stabilization of drug molecules through ion-pairing, microenvironmental pH modulation, and inhibition of recrystallization. Their integration into diverse oral formulations—including active pharmaceutical ingredient ionic liquids (API-ILs), lipid-based systems, and peptide carriers—has yielded promising pharmacokinetic improvements in preclinical models (Table 1). The following subsections synthesize the main mechanistic and formulation trends observed across oral IL systems. A mechanistic overview summarizing how ILs modulate solubility, permeability, stability, and nanoassembly formation across delivery routes is depicted in Figure 2.

**Table 1:**
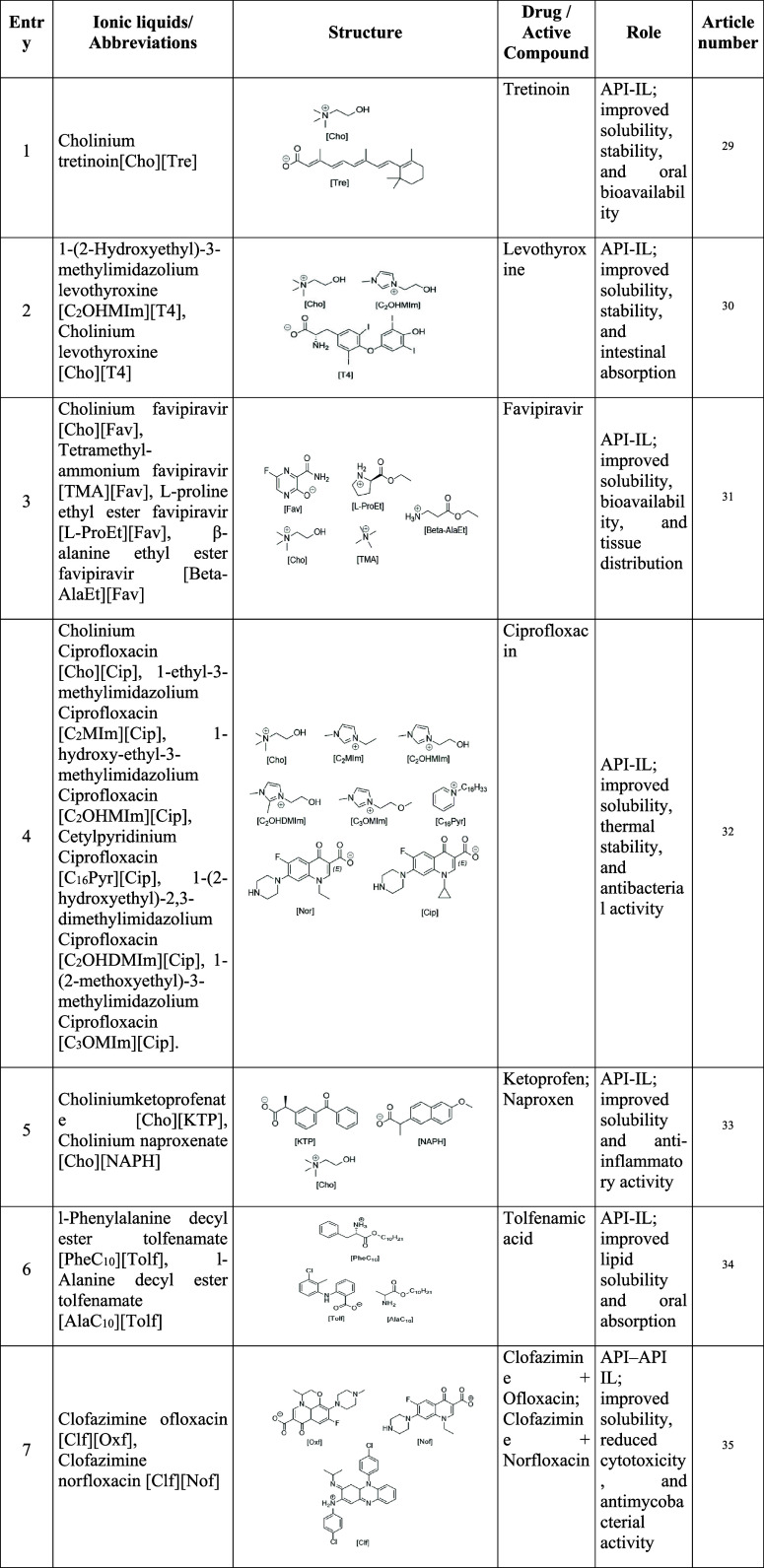

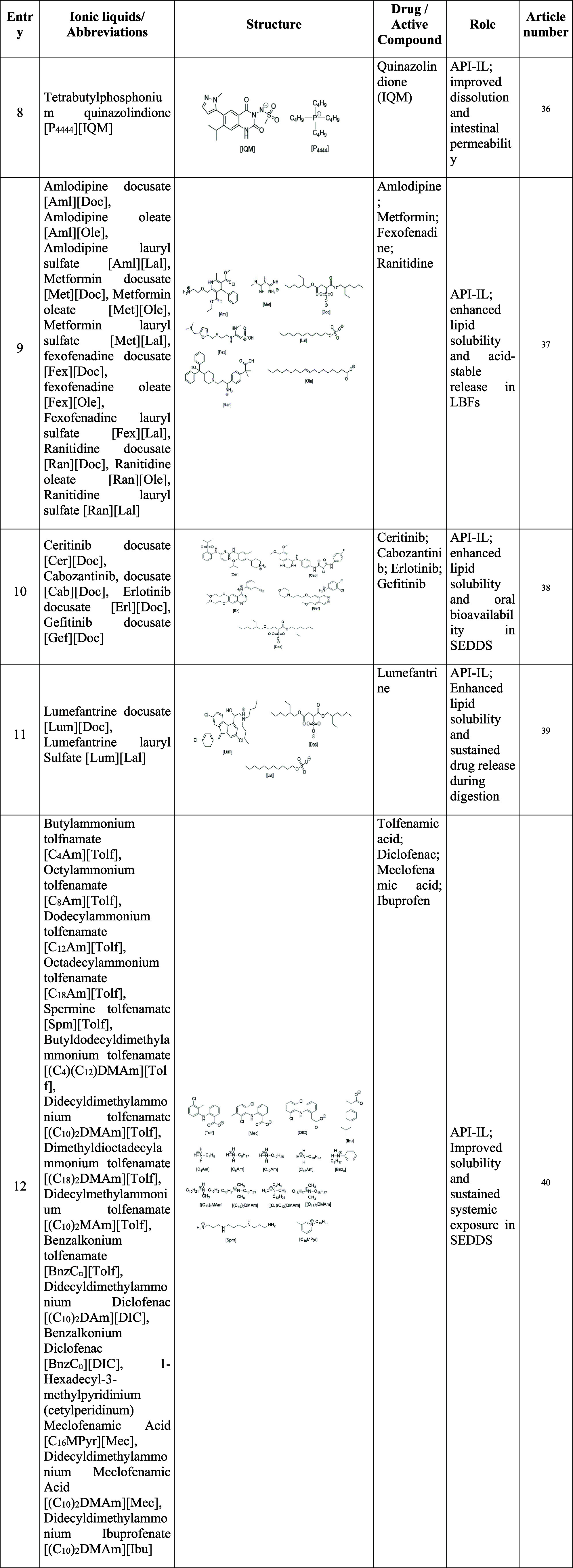

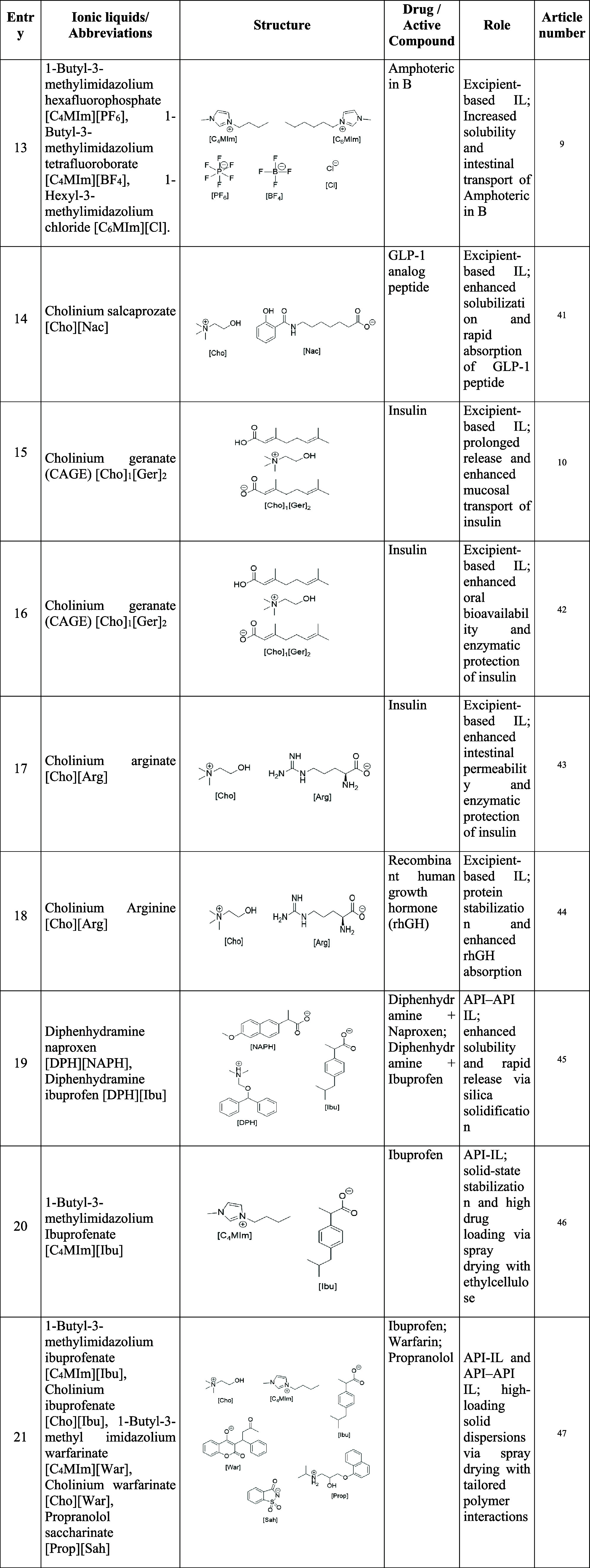

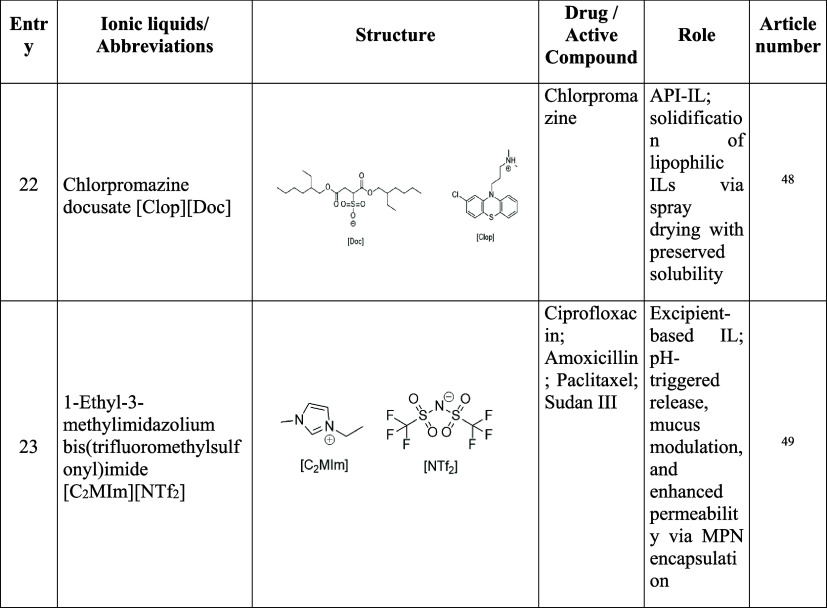
Summary of Ionic Liquids Investigated in Oral Pharmaceutical Formulations, Including Abbreviations, Chemical Structure, Active Compound, Functional Role, and Corresponding Reference Numbers

**Figure 2:**
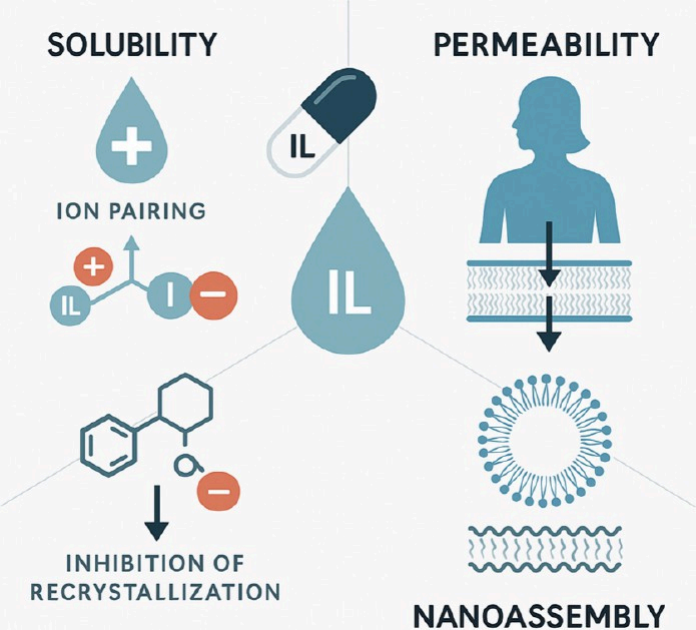
Mechanistic map illustrating the principal physicochemical and biological mechanisms by which ILs enhance pharmaceutical performance across multiple delivery routes. ILs improve the solubility through ion pairing and inhibition of drug recrystallization, stabilizing both hydrophilic and lipophilic APIs. They enhance the permeability by transiently modulating biological barriers and increasing drug diffusion across epithelial and mucosal tissues. ILs also drive nanoassembly, enabling the formation of micelles, vesicles, ionogels, and hybrid nanocarriers with a tunable release and structural properties. Together, these mechanisms underpin the multifunctional role of ILs as solubilizers, permeability enhancers, structural organizers, and bioactive formulation components.

#### 3.1.1 Solubility and Dissolution Enhancement

The transformation of poorly soluble drugs into API-ILs represents a versatile formulation strategy to overcome the dissolution and bioavailability limitations. By forming salts with tunable polarity, hydrogen-bonding capacity, and amphiphilic balance, ILs allow simultaneous optimization of solubility, stability, and absorption efficiency.

Hydrophilic and low-viscosity ILs such as cholinium- and imidazolium-based systems illustrate this ability to modulate the aqueous solubility. The IL form of tretinoin, [Cho]­[Tre], combined with [Cho]­[Ger], displayed a pronounced solubility enhancement—from 0.24 μg/mL (aqueous suspension) to 42.1 mg/mL—corresponding to *a* > 17,000-fold increase compared with the conventional suspension (Table 1, entry 1).^29^ Similarly, levothyroxine ILs prepared with [Cho] or [C_2_OHMIm] cations eliminated polymorphic transitions that reduce dissolution reproducibility of the crystalline drug, achieving greater diffusivity and biocompatibility at 37 °C (Table 1, entry 2).^30^ These studies show that hydrophilic ILs prevent crystallization and sustain supersaturation, leading to faster dissolution and improved systemic exposure.

Amphiphilic and biocompatible ILs bridge solubility enhancement with permeability improvement. Favipiravir ILs synthesized with [Cho], [TMA], or amino-acid-ester cations achieved up to 125-fold higher aqueous solubility than the parent drug and an enhanced intestinal absorption (Table 1, entry 3).^31^ Their amphiphilic character allows interactions with both hydrophilic and lipophilic domains, promoting dissolution and facilitating membrane diffusion. Comparable trends were observed for fluoroquinolone-based ILs such as [C_16_Pyr]­[Cip], which retained potent antimicrobial activity while showing superior solubility (Table 1, entry 4).^32^

Lipophilic and biodegradable ILs extend these advantages to lipid-based systems. Lipoamino-acid salts such as [AlaC_10_]­[Tolf] and [PheC_10_]­[Tolf] increased tolfenamic-acid solubility in simulated intestinal fluids and produced more than 3-fold higher maximum plasma concentration (*C*_max_) than the parent compound (Table 1, entry 6).^34^ Likewise, nonsteroidal anti-inflammatory drug (NSAID) ILs—[Cho]­[KTP] and [Cho]­[NAP]—showed 58-fold solubility improvements over their sodium salts (Table 1, entry 5).^33^ Enzyme-inhibition assays indicated that specific ion-pair combinations influence biological response: [Cho]­[NAP] exhibited stronger cytochrome c oxidase inhibition than [Na]­[NAP], whereas [Cho]­[KTP] displayed weaker inhibition, revealing the fine-tunability of IL-drug interactions.

Structurally optimized API–IL frameworks further illustrate the potential of a rational ion-pair design. API–API ILs such as [Clf]­[Oxf] and [Clf]­[Nof] enhanced DMSO solubility compared to the free drug, reduced macrophage cytotoxicity by ≈ 80%, and maintained antimycobacterial activity (Table 1, entry 7).^35^ The phosphonium-based IL [P_4,4,4,4_]­[IQM] showed a 700-fold faster dissolution rate than the crystalline parent compound and 3–5× greater transepithelial transport in Caco-2 monolayers without structural modification of the parent compound (Table 1, entry 8).^36^ Together, these examples demonstrate that ILs act as molecular solubilizers capable of stabilizing supersaturated phases and improving membrane transport while maintaining chemical integrity.

Across these systems, three dominant mechanisms underpin the solubility-enhancing behavior of ILs: (i) disruption of crystal lattices via ionic and hydrogen-bonding interactions; (ii) stabilization of metastable supersaturated drug phases that delay precipitation; and (iii) fine-tuning of solvent polarity and amphiphilicity to match the physicochemical environment of the API. Compared with conventional salt formation or cosolvent strategies, ILs provide a modular and multifunctional platform that couples solubility, stability, and permeability control within a single ion-pair architecture. Moreover, IL-based systems offer complementary molecular design flexibility compared with conventional salts or prodrugs, enabling modulation through ion pairing rather than covalent modification.

Nevertheless, challenges remain. Increased lipophilicity may elevate cytotoxicity, particularly for long-chain imidazolium and pyridinium cations, emphasizing the importance of biocompatible ion selection. Translating highly soluble ILs into solid dosage forms and clarifying their regulatory classification also pose formulation and approval barriers. Overall, ILs represent a tunable and powerful tool for improving drug solubility and dissolution provided that molecular design carefully balances physicochemical performance with biological safety.

#### 3.1.2 Lipid Compatibility and Gastrointestinal Solubilization

Lipid-based ILs constitute an effective strategy to enhance the oral bioavailability of poorly water-soluble APIs by integrating ionized drug forms into lipidic excipients. These systems—including self-emulsifying drug delivery systems (SEDDS), lipid-based formulations (LBFs), and soft gelatin capsules—combine the solubilizing capacity of lipids with the structural versatility of ILs, improving dissolution, maintaining supersaturation during digestion, and reducing precipitation under gastric conditions.

Lipophilic ion pairs such as docusate, lauryl sulfate, and oleate have been extensively employed to transform conventional salts into lipid-compatible ILs. For instance, amlodipine, fexofenadine, and metformin ILs prepared with these anions exhibited 20- to 30-fold higher solubility in PEG-400, Labrasol, and medium-chain triglycerides compared with their parent salts (Table 1, entry 9).^37^ These ILs maintained dispersion stability under acidic pH, preventing recrystallization and enabling efficient drug release upon intestinal transition. Similar behavior was reported for kinase-inhibitor ILs (gefitinib, cabozantinib, ceritinib), which formed low-melting salts (<100 °C) with docusate, achieving 3.5- to 5-fold higher oral bioavailability in SEDDS formulations relative to the unmodified drugs (Table 1, entry 10).^38^ These results demonstrate that lipophilic counterions act as “lipid anchors,” promoting solubilization in nonpolar excipients and protecting the API against gastric precipitation.

Amphiphilic lipid-ionic systems further reinforce solubilization across the digestive process. Lumefantrine ILs such as [Lum]­[Doc] and [Lum]­[Lal] increased solubility from 11 mg/g (free base) to 57 mg/g and maintained >60% solubilization during 4 h of simulated intestinal digestion, yielding a 1.8-fold increase in area under the concentration–time curve (AUC) (Table 1, entry 11).^39^ Likewise, tolfenamic-acid ILs paired with [(C_10_)_2_DMAm] achieved a 6-fold improvement in loading capacity (35 → 200 mg/g) and maintained >75% of the drug solubilized during dispersion and enzymatic digestion, sustaining plasma exposure for 48 h postdose (Table 1, entry 12).^40^ Such formulations highlight the capacity of ILs to synchronize drug solubilization and lipid digestion kinetics, sustaining colloidal stability and absorption under physiologically dynamic conditions.

Functional IL excipients also extend this principle to highly hydrophobic biopharmaceuticals. For amphotericin B (AmpB), incorporation of imidazolium-based ILs such as [C_4_MIm]­[BF_4_] and [C_6_MIm]­[Cl] within self-nanoemulsifying drug-delivery systems (SNEDDS) increased drug solubility from <0.5 to 1.69 mg/g, while enhancing transepithelial permeability ≈ 3.5-fold in Caco-2 monolayers (Table 1, entry 13).^9^ The formulations remained stable in simulated gastric and intestinal fluids for at least 4 weeks, underscoring the stabilizing role of ILs as cosolvents and interfacial modifiers in nanoemulsified carriers. This dual function—solubilization and interfacial stabilization—illustrates how ILs bridge the roles of surfactant and ion-pair modifier in complex lipid systems.

Across these systems, lipid-compatible ILs enhance bioavailability through three cooperative mechanisms: (i) ion pairing with long-chain or amphiphilic counterions that favor solubility in lipid excipients, (ii) maintenance of supersaturation during digestion by forming reversible ion pairs that suppress precipitation, and (iii) modulation of interfacial tension to stabilize self-emulsifying or nanoemulsion structures. Compared to traditional lipid formulations requiring additional surfactants or pH modifiers, ILs achieve equivalent or superior performance using fewer excipients and offering thermodynamic rather than kinetic stabilization.

However, recurring challenges persist. Highly lipophilic ions may induce phase separation or reduce drug release rates, while the liquid nature of many ILs complicates solid-dose conversion and scale-up. Additionally, the metabolic fate of ionic counterions under gastrointestinal conditions has remained insufficiently characterized. Therefore, while IL-lipid hybrids present a promising route to prolong solubilization and enhance absorption, their translation demands careful optimization of the ion structure, lipid compatibility, and formulation stability.

#### 3.1.3 Stabilization and Permeability Control for Peptides

Peptide delivery through the oral route remains challenging due to rapid enzymatic degradation, poor epithelial permeability, and dilution during gastric transit. IL-based systems address these limitations through three recurrent mechanistic strategies: (i) stabilization in liquid or semisolid environments, (ii) enhanced epithelial transport, and (iii) enzymatic and mucus-barrier modulation.

##### 3.1.3.1 Peptide Stabilization and High-Load Solubilization

ILs can maintain peptides in chemically stable, high-concentration liquid phases, which limit precipitation and improve luminal persistence. For example, [Cho]­[Nac], derived from salcaprozic acid, solubilized 675 mg/mL of salcaprozate—over twice the solubility achievable in conventional aqueous formulations—and preserved the GLP-1 analog for ≥3 weeks at both 22 and 4 °C (Table 1, entry 14).^41^ This stabilization enabled fast absorption in rats and dogs and short-term controlled release, illustrating how IL-mediated peptide solubilization supports rapid onset formulations.

##### 3.1.3.2 Enhancing Permeability: Mucoadhesive Ionogels and Enteric Systems

The cholinium geranate IL (CAGE) represents a widely studied permeability-enhancing excipient. Incorporated into PVA-based ionogels, CAGE increased transepithelial transport of insulin by ≈30% in Caco-2/HT29-MTX cocultures and supported sustained release for up to 5 h, with improved mucosal compatibility relative to CAGE solution (Figure 3) (Table 1, entry 15).^10^ Enteric-coated insulin–CAGE capsules further achieved 45% glucose reduction over 10 h and 51–66% relative bioavailability (Table 1, entry 16),^42^ approaching subcutaneous administration. Mechanistic studies consistently show transient transepithelial electrical resistance (TEER) reduction (≈50%), tight-junction modulation, and 13-fold increases in paracellular transport, explaining the marked improvement in peptide uptake.

**Figure 3:**
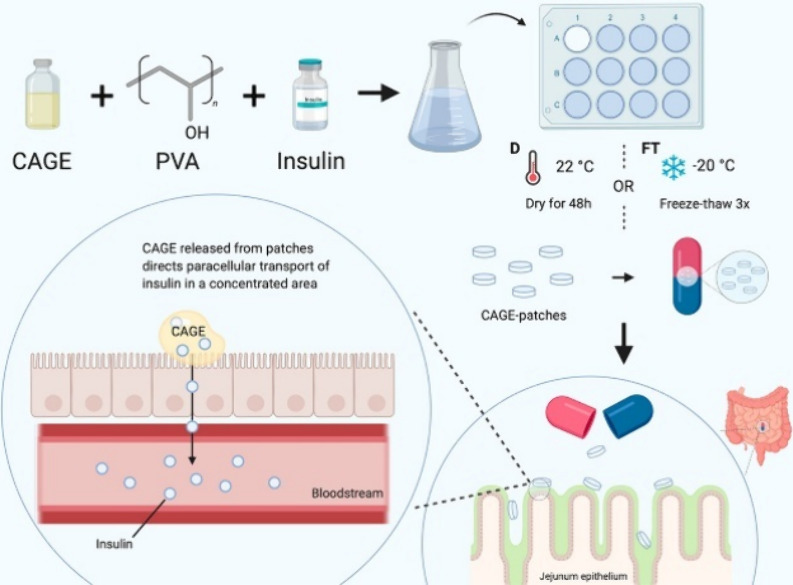
Fabrication of CAGE-patches for oral delivery. CAGE-patch ionogels were fabricated by combining CAGE, PVA, and insulin into a mixture. Mixtures were poured into cell culture plates and either left to dry for 48 h to make D-CAGE-patches or subjected to repeated freeze–thaw cycles for FT-CAGE-patches. In the potential clinical setting, patches would be loaded into enteric-coated pills that dissolve and release into the small intestine. Schematic created with Biorender.com. Reprinted with permission from ref 10. Copyright 2023 American Chemical Society.

##### 3.1.3.3 Enzymatic Protection and Transport via Nanoparticulate IL Systems

Nanoparticle systems incorporating [Cho]­[Arg] provide complementary benefits by shielding peptides from gastric and intestinal proteases. In PLGA–chitosan systems modified with vitamin B_12_, [Cho]­[Arg] protected insulin from pepsin and trypsin, decreased TEER, enhanced permeability, and produced 3-fold higher insulin absorption with 57% glycemic reduction in diabetic mice (Figure 4) (Table 1, entry 17).^43^ Co-formulation of [Cho]­[Arg] with DCA enabled segmental absorption of recombinant human growth hormone (rhGH) and maintained protein integrity under acidic and neutral pH, yielding 86% relative bioavailability in rodents and significant pharmacodynamic benefits (Table 1, entry 18).^44^

**Figure 4:**
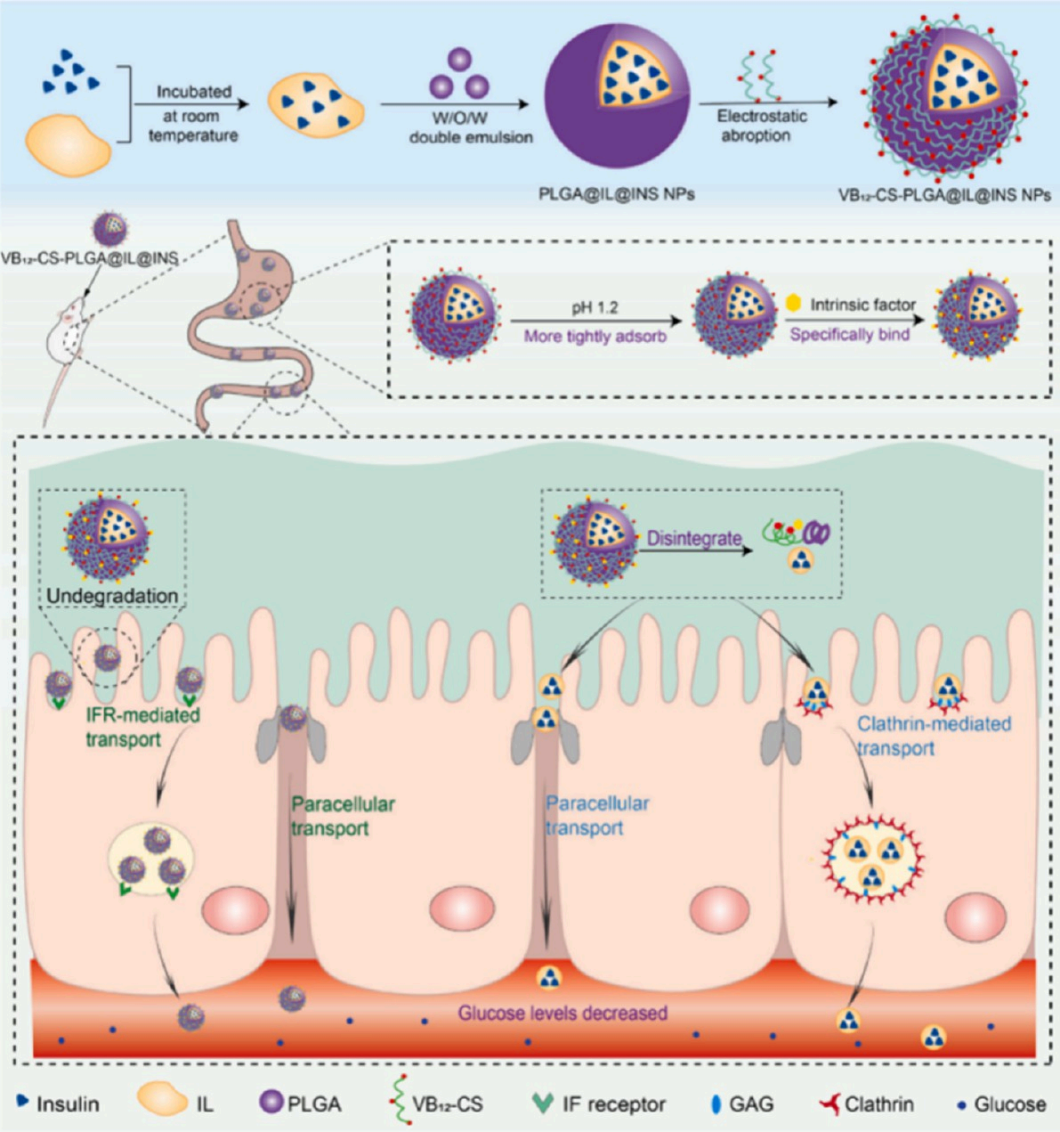
Schematic diagram of the synthesis of “ternary mutual-assist” nanodelivery system VB_12_-CS-PLGA@IL NPs and improving oral insulin bioavailability. Reprinted with permission from ref 43. Copyright 2023 Elsevier B.V.

##### 3.1.3.4 Cross-Cutting Mechanistic Themes

Across formulations, ILs improve peptide delivery through common, reproducible mechanisms: (i) Solubilization and stabilization in high-load liquid IL environments (e.g., [Cho]­[Nac]); (ii) modulation of epithelial barriers, including mucus thinning, tight-junction opening, and reduced TEER (CAGE, [Cho]­[Arg]); (iii) protection from proteolysis, preserving 25–75% of intact peptide after hours of enzymatic exposure—substantially higher than buffer-based controls; and (iv) facilitation of paracellular and transcellular pathways, enabling sustained systemic exposure without observed mucosal damage. Collectively, these IL-mediated strategies provide a coherent framework for enhancing oral peptide delivery by simultaneously addressing solubility, permeability, and enzymatic stability—parameters rarely improved in tandem using conventional excipients.

#### 3.1.4 Solid-State Engineering and Hybrid Formulations

Spray drying and adsorption onto porous carriers provide effective strategies to convert viscous or liquid API-ILs into free-flowing solids, enabling high-loading oral dosage forms that maintain the solubilization capacity of ILs while improving their manufacturability. Across studies, two dominant approaches emerge: (i) adsorption of API-ILs onto high-surface-area matrices to improve handling and dissolution, and (ii) hybrid solidification using immiscible or partially miscible polymers to stabilize amorphous IL dispersions.

##### 3.1.4.1 Adsorption-Based Systems

Protic ILs such as [DPH]­[IBU] and [DPH]­[NAP] achieved 8–10-fold higher solubility than their free acid forms, but their high viscosity impaired capsule performance. Adsorbing these ILs onto mesoporous silica converted them into free-flowing powders capable of releasing ≈90% of the drug within 15 min under simulated gastric conditions (Table 1, entry 19).^45^ This reflects a recurring trend: surface confinement mitigates the rheological limitations of viscous ILs, improving the wettability and dissolution without altering their ionic structure. These systems consistently show a faster Tmax and improved predicted bioavailability, underscoring the benefit of mesoporous carriers for highly viscous API-ILs.

##### 3.1.4.2 Polymer–IL Hybrid Matrices

Encapsulation of ILs such as [C_4_MIm]­[Ibu] or [Cho]­[War] in ethylcellulose produces amorphous powders with high drug loading (≈75% w/w) and long-term stability exceeding two years (Table 1, entries 20–21).^46,47^ These examples highlight a second mechanistic theme: immiscibility-driven physical entrapment, where the IL phase remains molecularly separate yet stabilized within a polymeric scaffold. This retains the IL’s solubilizing power while preventing crystallization or phase separation. In formulations where IL–polymer miscibility is higher, such as [Prop]­[Sah] with maltodextrin, molecular amorphous dispersions form instead, showing how polymer–IL interaction tuning can modulate the solid-state structure and release behavior.

##### 3.1.4.3 Lipid/Polymer Hybrid Systems

For highly lipophilic ILs such as [Clop]­[Doc], combining lipid-based formulations with spray drying yielded solid powders capable of generating <270 nm droplets during digestion, maintaining solubilization far better than chlorpromazine base and HCl forms (Table 1, entry 22).^48^ These hybrid systems illustrate a third mechanistic advantage: ILs compatible with lipid excipients preserve supersaturation during gastrointestinal digestion, enabling absorption-enhancing colloidal structures that traditional salts fail to access.

##### 3.1.4.4 Cross-Cutting Advantages and Challenges

Collectively, these approaches show that solid-state engineering allows ILs to retain their solubilization efficiency while overcoming handling and dosing limitations and addressing viscosity, flowability, and hygroscopicity. However, recurring formulation challenges include IL hygroscopicity, high glass transition variability, potential phase immiscibility, and the need for excipients that balance stabilization with redispersion. Mechanistically, the success of these systems relies on confining ILs within porous, polymeric, or lipid-solid matrices that preserve amorphous character, prevent aggregation, and ensure rapid reconstitution during digestion, making them promising routes for the high-loading oral delivery of IL-based APIs.

#### 3.1.5 Stimuli-Responsive Systems and Mechanistic Insights

Stimuli-responsive IL systems introduce an added design dimension to oral delivery by coupling environmental triggers—typically pH—with the solubilization and permeability-enhancing capabilities of ILs. A representative example is the IL@MPN platform, in which [C_2_MIm]­[NTf_2_] is encapsulated within a Fe­(III)–quercetin metal–phenolic network (Figure 5) (Table 1, entry 23).^49^ These microcapsules remained structurally stable at neutral pH but released >98% of their cargo within 30 min under gastric acidity (pH 2.0), demonstrating precise pH-triggered release. In mice, the IL@metal-phenolic network (MPN) enhanced intestinal penetration of fluorescent tracers and paclitaxel relative to PBS controls, confirming that acidic activation facilitates mucosal permeation while preserving biocompatibility at relevant doses.

**Figure 5:**
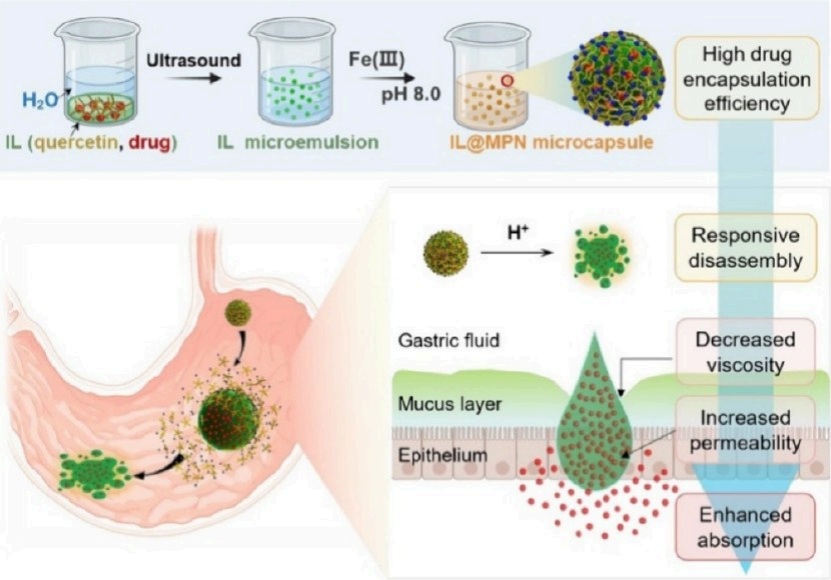
Preparation and working mechanism of the proposed IL@MPN microcapsule oral delivery system. Reprinted with permission from ref 49. Copyright 2022 American Chemical Society.

Mechanistically, these systems integrate two complementary functions: (i) Stimulus-driven release, governed by the disassembly of the phenolic network in acidic media; and (ii) IL-mediated permeability enhancement, as [C_2_MIm]­[NTf_2_] decreased mucus viscosity and increased transcellular transport in Caco-2 cells without disrupting epithelial integrity.

Across studies, the IL structure emerges as a determinant of mechanistic behavior. Long-chain imidazolium ILs can transiently disrupt lipid bilayers and increase membrane fluidity, resulting in strong permeation enhancement, but at the cost of higher cytotoxicity. In contrast, cholinium-based and amino-acid-derived ILs offer a more favorable safety profile, promoting moderate permeability enhancement while maintaining epithelial integrity and compatibility with polymeric matrices. This divergence highlights the need to balance amphiphilicity, biocompatibility, and formulation processability in the design of orally administered IL systems.

Overall, stimuli-responsive IL platforms demonstrate how triggered release, mucus modulation, and fine-tuned epithelial interactions can be combined in a single system. These mechanistic features broaden the design space of oral delivery technologies and support the development of IL-enabled formulations that are both effective and translatable to clinical settings.

### 3.2 Injectable Formulations

The parenteral route enables precise dose control and rapid systemic exposure but imposes stringent requirements on solvent safety, isotonicity, biocompatibility, and formulation stability. Within this context, ILs have been employed to solubilize hydrophobic drugs, stabilize monomeric species, and engineer colloidal architectures, such as nanoemulsions, nanoparticles, and depot matrices (Table 2). These systems leverage the amphiphilic and tunable nature of ILs to address key limitations in injectables, including poor aqueous solubility, aggregation-induced loss of potency, and the need for controlled or localized drug release. The studies summarized below highlight recurring design strategies and mechanistic principles guiding IL-based injectable formulations.

**Table 2:**
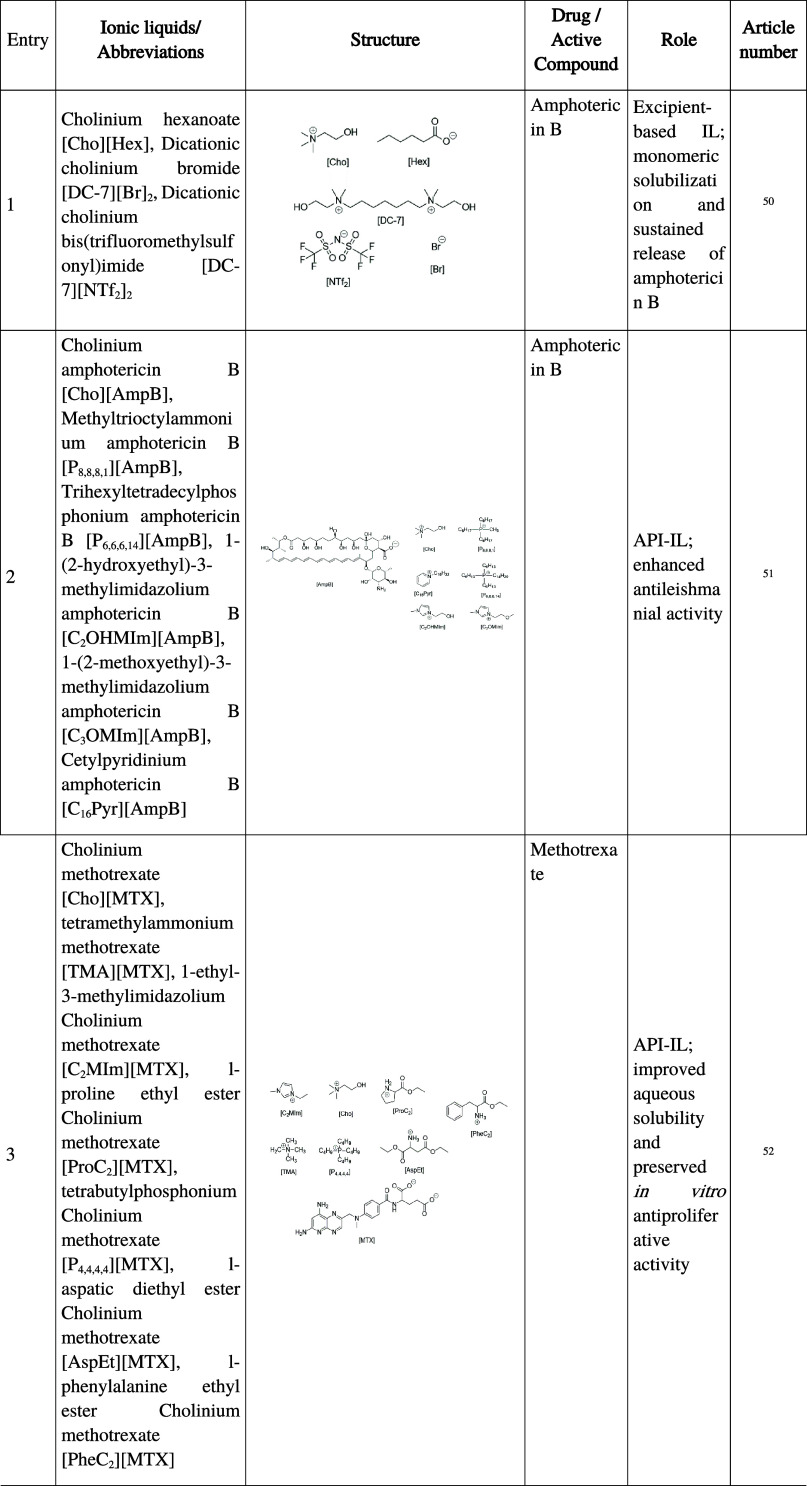

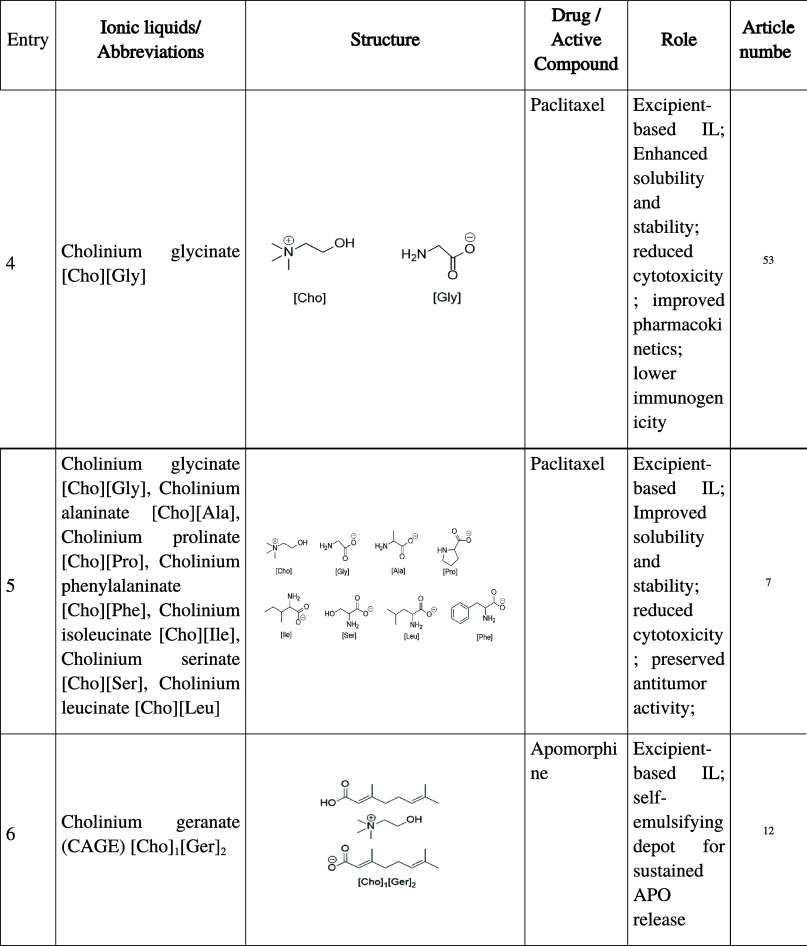

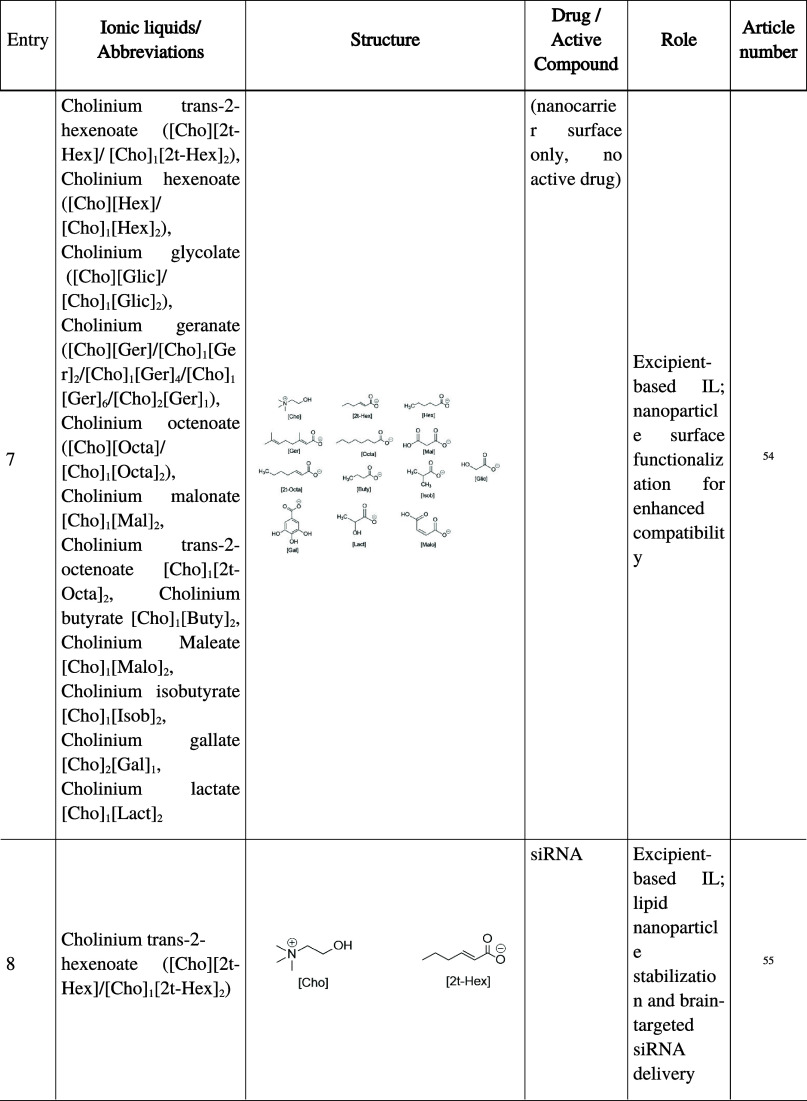

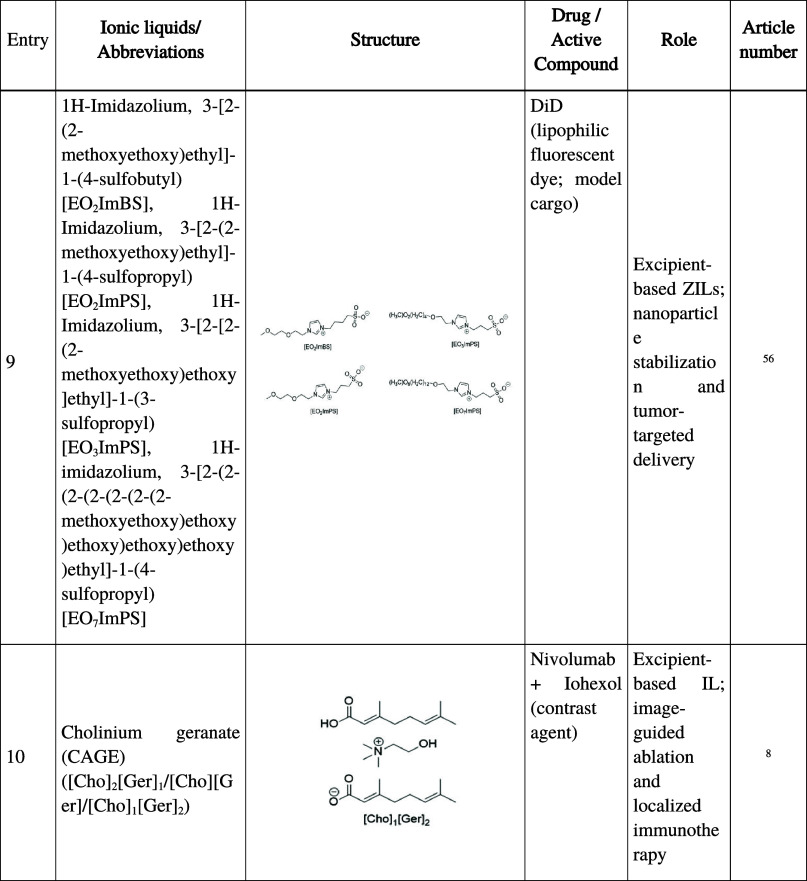
Summary of Ionic Liquids Investigated in Injectable Pharmaceutical Formulations, Including Abbreviations, Chemical Structure, Active Compound, Functional Role, and Corresponding Reference Numbers

#### 3.2.1 Solubilization and Stabilization of Hydrophobic Drugs

A central motivation for incorporating ILs into injectable formulations is their ability to replace toxic solubilizers—such as Cremophor EL—while simultaneously improving the solubilization and stabilization of hydrophobic or aggregation-prone APIs. Across diverse drug classes, IL-based systems consistently enable higher monomeric drug loading, reduced excipient toxicity, and improved colloidal stability relative to conventional vehicles (Table 2, entries 1–4).

##### 3.2.1.1 ILs as Vehicle Components for Monomeric Drug Solubilization

Hydrophobic ILs such as [Cho]­[Hex] and the dicationic IL [DC-7]­[NTf_2_]_2_ generate nanoemulsion environments capable of maintaining notoriously aggregation-prone amphotericin B (AmB) in its monomeric state at clinically relevant concentrations (up to 6 mg/mL, vs <0.1 mg/mL in water). Preventing aggregation directly addresses the dominant mechanism of AmB toxicity. These IL nanoemulsions showed <1% hemolysis, sustained release over 24 h, and complete zebrafish embryo survival at 889 μM—evidence of improved compatibility compared to Cremophor EL micelles (Table 2, entry 1).^50^ Collectively, these findings illustrate how IL polarity and chain length can be tuned to avoid aggregation while stabilizing colloidal dispersions suitable for parenteral use.

##### 3.2.1.2 API–IL Transformation for Improved Solubility and Retained Activity

An alternative strategy converts the active pharmaceutical ingredient itself into an IL. Phosphonium-, cholinium-, and imidazolium-based salts of AmB exhibited comparable or enhanced antiparasitic activity relative to the parent drug, with [P_6,6,6,14_]­[AmB] reaching an IC_50_ of 61.4 nM versus 86.6 nM for free AmB (Table 2, entry 2).^51^ While these results validate the biological functionality of API-ILs, the absence of stability, hemocompatibility, or in vivo pharmacokinetic data limits the current assessment of their parenteral applicability. This highlights a broader challenge for injectable ILs: biological activity does not necessarily predict systemic tolerability, underscoring the need for toxicity-resolved optimization.

##### 3.2.1.3 Enhancing Solubility of Small-Molecule Chemotherapeutics

Ionic transfer to cholinium or amino-acid ester cations dramatically improved the aqueous solubility of methotrexate (MTX), reaching up to 5280-fold increases relative to the free drug and approximately 2× versus its sodium salt (Table 2, entry 3).^52^ The resulting ILs remained amorphous and retained antiproliferative activity in HeLa cells, indicating that ion pairing improves dissolution while preserving cytotoxic function. Toxicity trends varied with the cation structure, suggesting that future MTX-IL designs should balance enhanced solubility with predictable pharmacokinetic and safety profiles.

##### 3.2.1.4 Replacing Cremophor EL in Paclitaxel Formulations

The most clinically relevant application comes from paclitaxel (PTX) formulations using cholinium–amino acid ILs (Table 2, entries 4–5).^7,53^ ILs such as [Cho]­[Gly] increased PTX solubility to 22.34 mg/mL (vs <0.5 mg/mL in Cremophor EL), maintained chemical stability for at least three months, and exhibited markedly reduced nonspecific cytotoxicity in HeLa and THP-1 cells. In B16F10 tumor-bearing mice, IL-PTX systems preserved antitumor efficacy while significantly reducing hypersensitivity reactions and lowering serum histamine and SC5b-9 levels—clear indicators of improved systemic safety. These outcomes demonstrate how biocompatible ILs can address both solubility bottlenecks and excipient-induced toxicity, which remains a major limitation of current PTX injections.

##### 3.2.1.5 Overall Trends and Mechanistic Insights

Across these examples, injectable IL systems improve performance through two recurring mechanisms: (i) molecular solubilization and aggregation control, driven by IL hydrophobicity, hydrogen-bond donor/acceptor balance, and amphiphilicity; and (ii) reduced vehicle toxicity, especially when replacing surfactants or cosolvents that trigger hemolysis or hypersensitivity. However, translation requires attention to the IL biodegradability, isotonicity, and systemic clearance. While ILs clearly enhance solubility and biological performance, long-term safety and pharmacokinetic profiling remain underexplored for many candidate systems.

#### 3.2.2 Depot-Forming and Sustained-Release Systems

Beyond solubilization, ILs have been incorporated into injectable depot platforms to achieve localized, sustained drug release following subcutaneous administration. A representative example is the self-emulsifying apomorphine (APO)-releasing therapeutic (SEAPORT), which combines [Cho]­[Ger], *N*-methylpyrrolidone, and PEG 3350 to form a self-emulsifying IL-based depot (Figure 6) (Table 2, entry 6).^12^ Upon injection, the formulation spontaneously emulsifies in situ, generating a drug reservoir that dissolves APO at 30 mg/mL (vs <2 mg/mL in aqueous formulations) and maintains >90% stability for at least 7 days across 4–37 °C.

**Figure 6:**
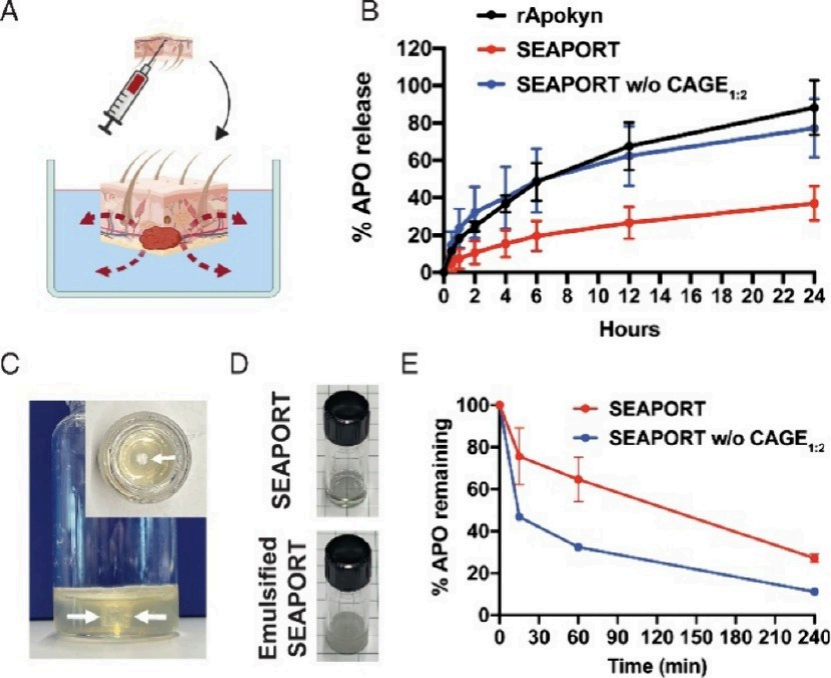
Ex vivo diffusion of APO. (A) Schematic illustration of ex vivo apparatus for APO release study from harvested rat skin. (B) Percent release of APO into saline from harvested rat skin subcutaneously injected with 50 μL of rApokyn, SEAPORT, and SEAPORT w/o CAGE_1:2_, as determined by LC-MS (*n* = 5, mean ± SD). (C) Photograph images of 36 wt %/vol agarose gel prepared in 20 mL glass scintillation vial (white arrows, hollow channel at the center of agarose gel). (D) Photograph image of opaque, emulsified SEAPORT after incubation in the agarose gel. (E) Percent of APO remaining at the center of agarose gel after incubation at 37 °C for specified time point, determined by LC-MS (*n* = 3, mean ± SD). Reprinted with permission from ref 12. Copyright 2022 the Author(s). Published by PNAS under CC BY-NC-ND 4.0.

In vivo, SEAPORT significantly extended systemic exposure in rats and pigs to 24–48 h, whereas the commercial formulation Apokyn achieved less than 8 h. The IL depot increased both AUC and time above the minimum effective concentration by approximately 2–3-fold, without inducing local irritation or histological damage. These safety findings are consistent with the inherent biocompatibility and antioxidative properties of cholinium-based ILs, which suppress injection-site inflammation compared to conventional cosolvent depots.

Collectively, these results demonstrate that ILs can function as biocompatible structuring matrices that promote in situ depot formation, stabilize labile drugs, and provide prolonged and predictable absorption. Such attributes highlight ILs as promising excipients for long-acting injectable (LAI) formulations aimed at chronic therapies requiring sustained plasma concentrations and a reduced dosing frequency.

#### 3.2.3 ILs as Surface Modifiers for Nanocarriers

ILs are also employed as functional coatings to tune nanocarrier interfaces and prolong the systemic circulation. PLGA nanoparticles modified with [Cho]­[Hex] resisted protein-corona formation for 96 min in mouse serum—approximately four times longer than unmodified PLGA (Table 2, entry 7)—and exhibited only 1.5% red-blood-cell adhesion, confirming hemocompatibility and suitability for intravenous administration.^54^

Geranic-acid ILs ([Cho]­[2t-Hex], [Cho]_1_[2t-Hex]_2_) incorporated into lipid nanoparticles for siRNA delivery (Table 2, entry 8) increased colloidal stability, reduced protein adsorption, and enhanced uptake by brain endothelial and motor-neuron cells while avoiding cytotoxicity.^55^ ILs were effective both as surface coatings and as components within the lipid matrix, underscoring their versatility for CNS-targeted nanocarriers.

Extending this concept, zwitterionic imidazolium ILs (ZILs) grafted onto PEG–PLGA nanoparticles (Table 2, entry 9) produced colloidally stable dispersions for up to 3 weeks and decreased immunoglobulin adsorption by ≈70% relative to unmodified controls.^56^ In murine whole blood, 2–3% of ZIL-NPs adhered reversibly to erythrocytes, suggesting potential for red-blood-cell hitchhiking to prolong circulation time. ZILs with longer alkyl chains also promoted preferential uptake by tumor cells over normal cells without detectable toxicity.

Collectively, these studies demonstrate that IL surface engineering can balance colloidal stability, stealth behavior, and targeted cellular uptake—highlighting ILs as modular tools for optimizing parenteral nanocarrier performance.

#### 3.2.4 ILs for Image-Guided and Localized Injectable Therapy

ILs have also been exploited for localized image-guided delivery. The Prostate Ablation and Drug Delivery Agent (PADA) system, composed of [Cho]_2_[Ger]_1_, iohexol (contrast agent), and nivolumab, combined therapeutic and diagnostic functions (Table 2, entry 10).^8^ Ex vivo and in vivo studies in porcine, canine, and TRAMP-C2 murine models demonstrated that PADA enabled localized ablation, controlled drug diffusion, and prolonged retention at the injection site, with no systemic toxicity. The inclusion of [Cho]_2_[Ger]_1_ provided both solvent functionality and intrinsic antimicrobial and anti-inflammatory activities, contributing to tissue compatibility. This system exemplifies how ILs can serve as multifunctional vehicles capable of delivering simultaneous therapy and imaging in localized interventions.

#### 3.2.5 Mechanistic Themes in IL-Based Injectable Systems

Across injectable systems, three main mechanistic trends emerge:

1. **Solubilization and stabilization:** ILs outperform conventional surfactants by forming stabilizing ion pairs that maintain APIs in monomeric or molecularly dispersed states, thereby preventing aggregation-driven toxicity and enabling high-concentration formulations.
2. **Controlled release and depot formation:** Amphiphilic ILs, such as [Cho]­[Ger], act as structural matrices that spontaneously organize into in situ depots after injection, supporting sustained drug release and prolonged systemic exposure relative to aqueous or micellar formulations.
3. **Surface modification of nanocarriers:** Zwitterionic and choline-based ILs modulate surface charge, protein adsorption, and interfacial hydration layers, conferring stealth properties, improving colloidal stability, and enhancing targeted uptake.

Comparatively, ILs intended for parenteral delivery must satisfy more stringent purity, hemocompatibility, and toxicity requirements than those used in oral systems (Section 3.1). While oral ILs primarily address solubility and permeability barriers, injectable ILs must additionally ensure isotonicity, minimal immunogenicity, and long-term colloidal stability. Their ability to reduce or eliminate reliance on excipients such as Cremophor EL underscores their translational potential; however, comprehensive toxicological and pharmacokinetic evaluations remain essential before clinical implementation.

### 3.3 Topical and Transdermal Formulations

Topical and transdermal platforms require materials capable of traversing the stratum corneum while maintaining biocompatibility and formulation stability. Within this context, ILs function not merely as excipients but as multifunctional agents—enhancing drug solubility and skin permeability, structuring self-assembled reservoirs for controlled release, and in some cases providing intrinsic pharmacological or stimuli-responsive behavior (Table 3). These combined roles enable improved partitioning into the stratum corneum and the more precise modulation of drug deposition and retention.

**Table 3:**
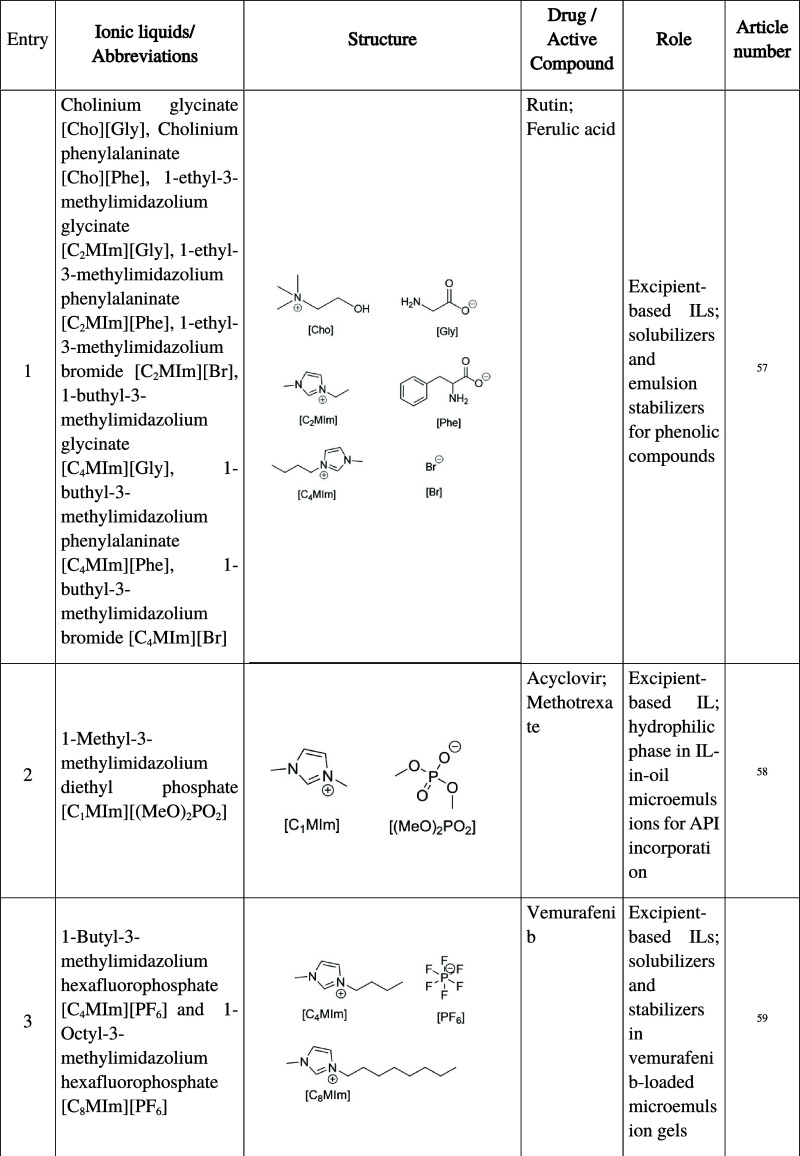

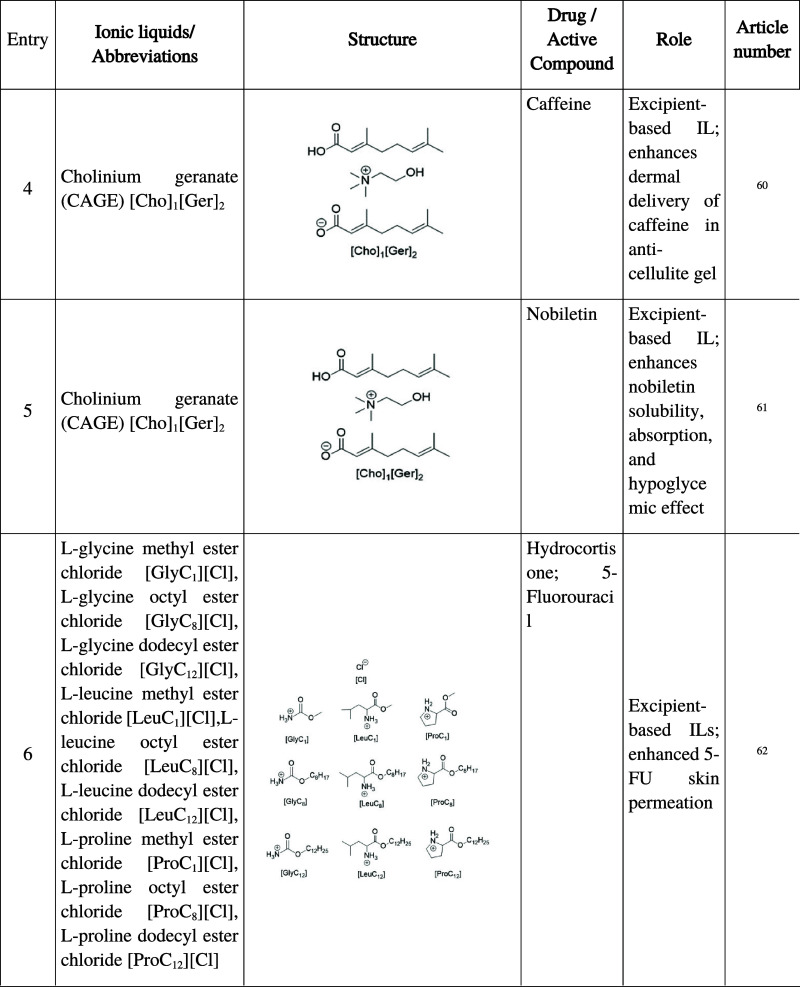

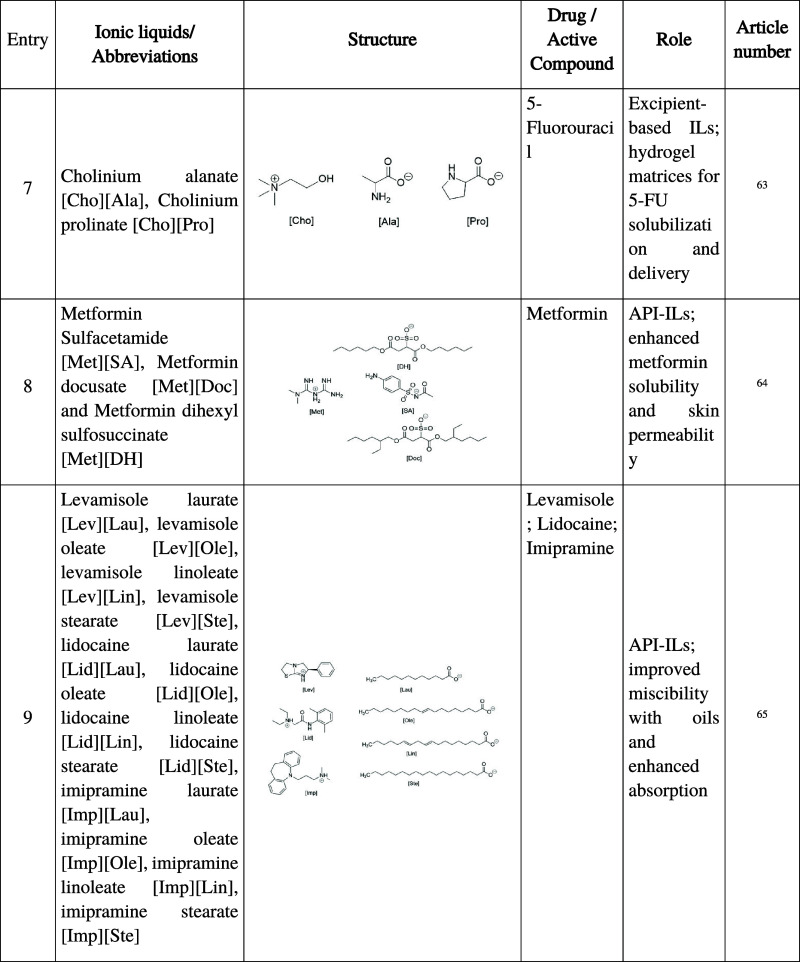

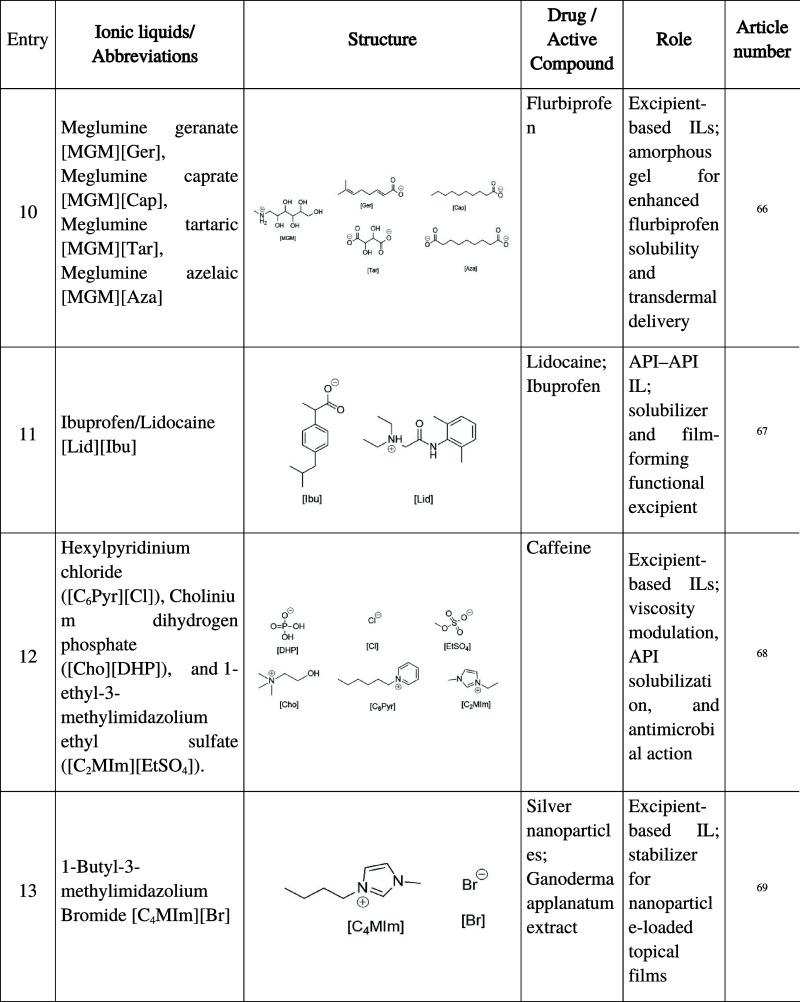

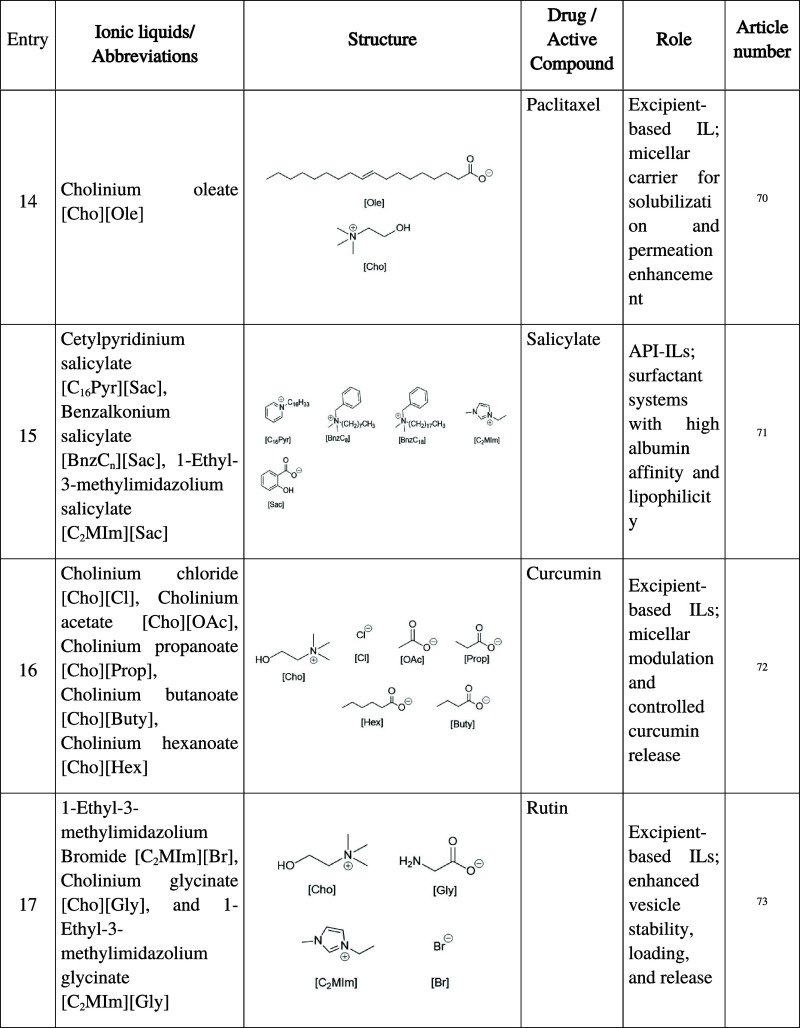

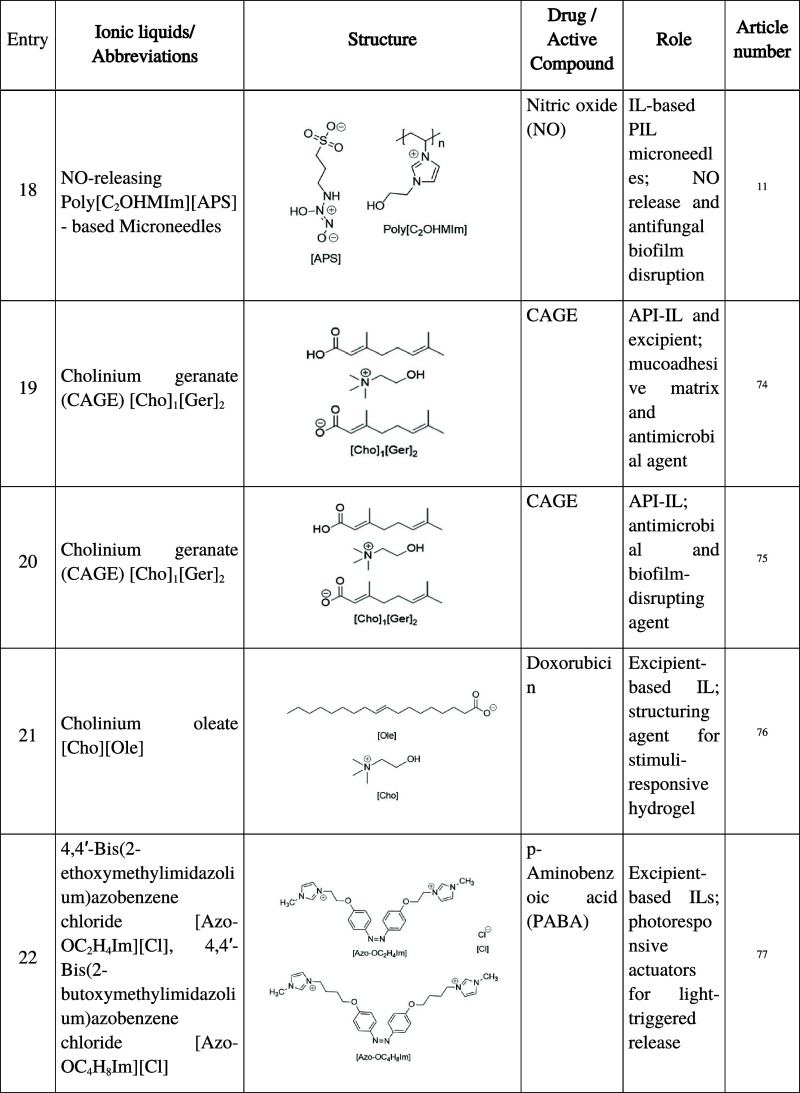

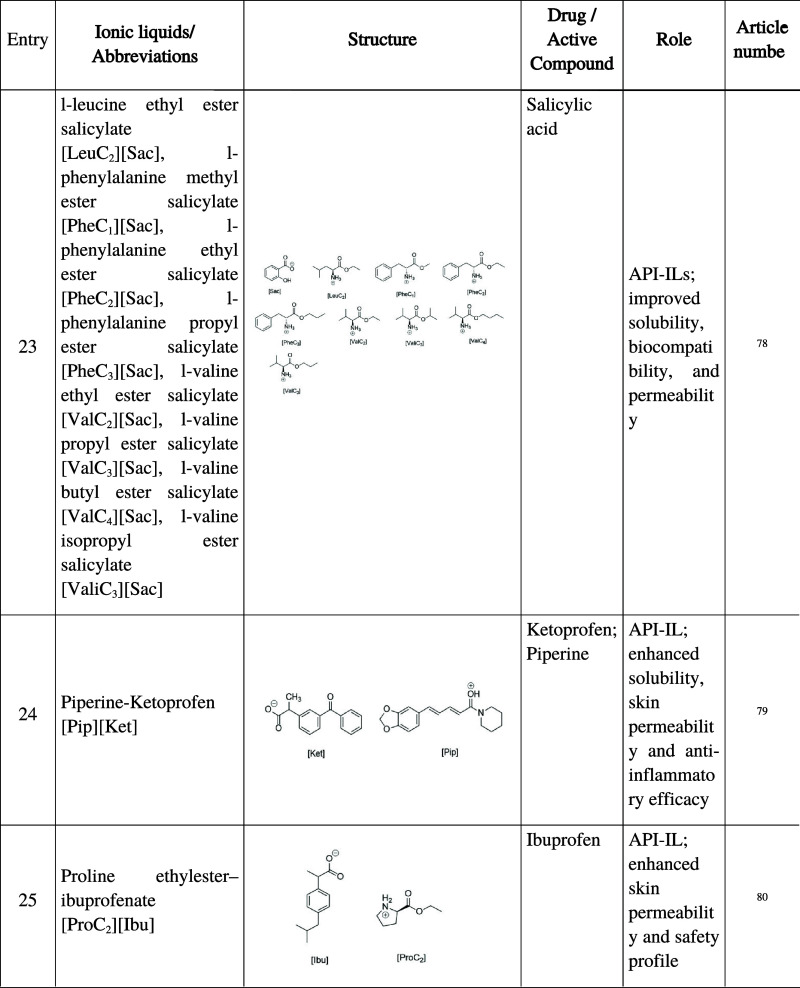

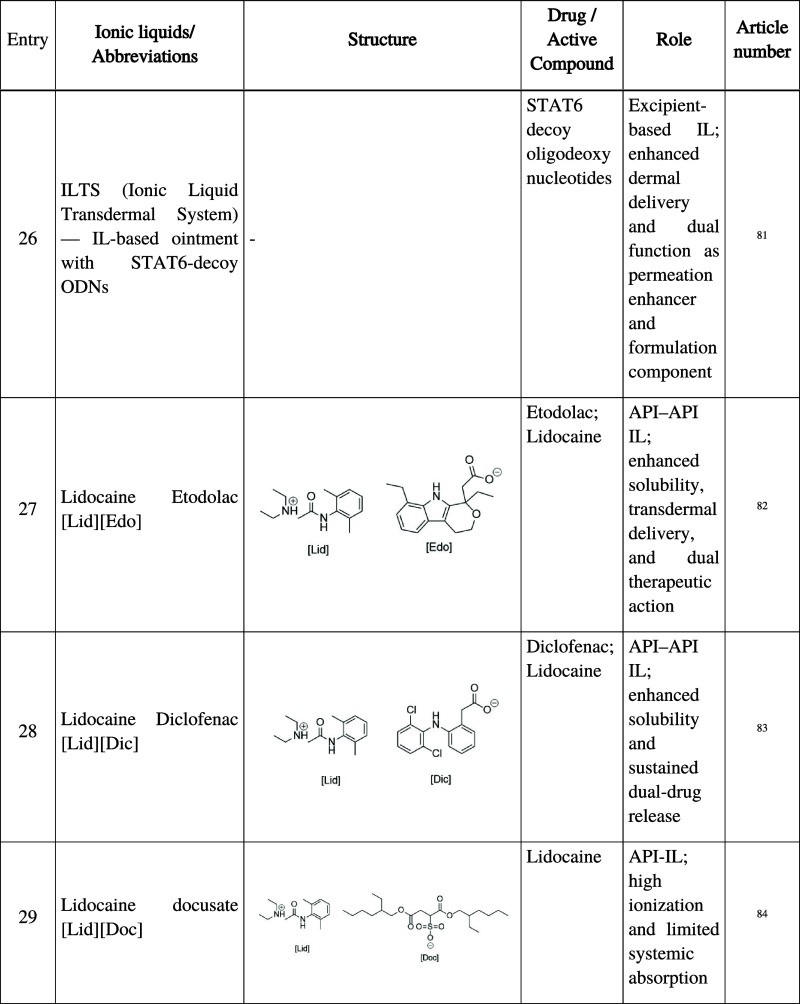

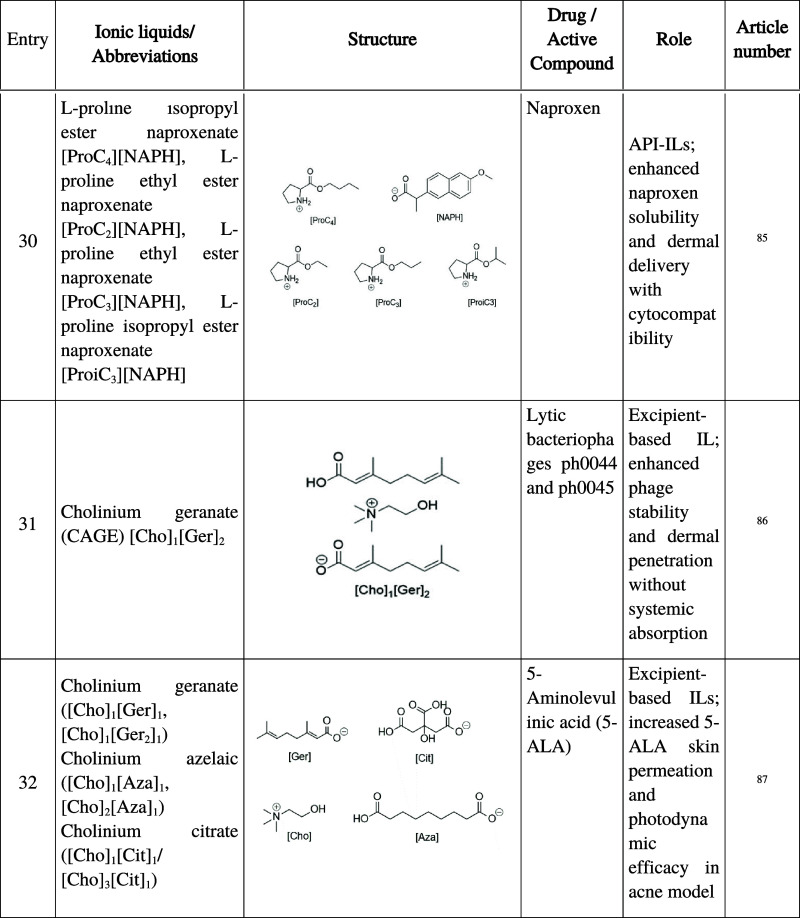

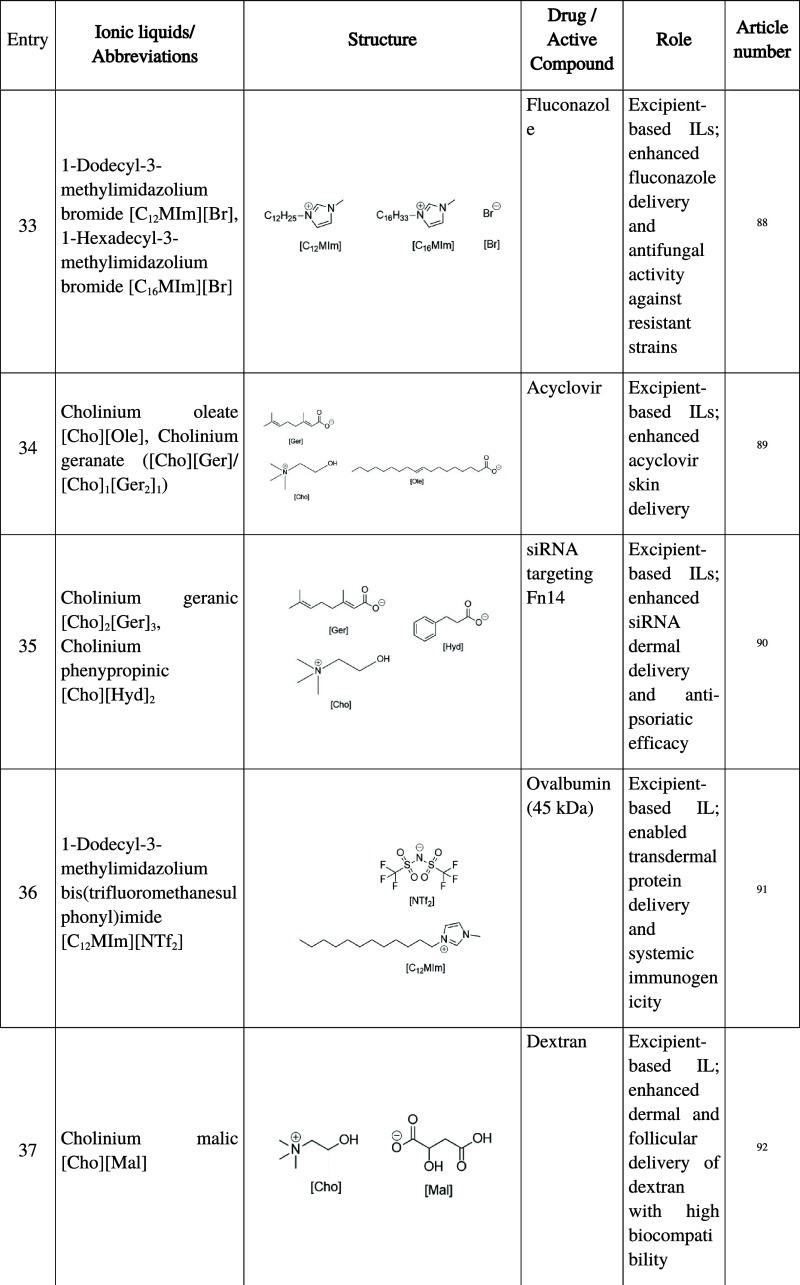

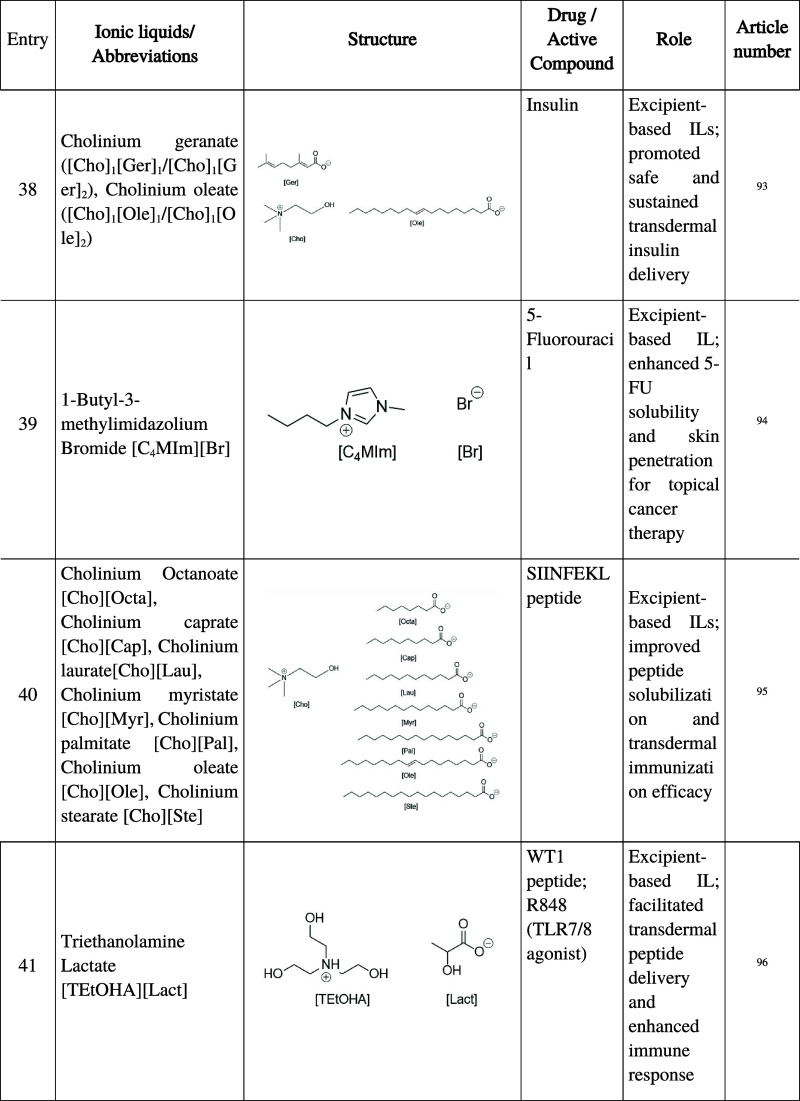
Summary of Ionic Liquids Employed in Topical and Transdermal Pharmaceutical Formulations, Including Abbreviations, Chemical Structure, Active Compound, Functional Role, and Corresponding Reference Numbers

#### 3.3.1 Solubilization and Permeation Enhancement

Topical and transdermal delivery demands excipients that enhance solubility and traverse the stratum corneum while preserving biocompatibility. Hydrophilic ILs derived from choline or imidazolium cations paired with amino-acid anions have proven to be highly effective in this role, markedly increasing solubility and cutaneous penetration of poorly soluble APIs while maintaining cytocompatibility. For example, [Cho]­[Gly] and [C_4_MIm]­[Gly] (Table 3, entry 1) enhanced rutin and ferulic-acid solubility by several orders of magnitude relative to water, while preserving full HaCaT viability at ≤0.5% v/v.^57^ These ILs also functioned as emulsion stabilizers and viscosity modifiers, improving the physical architecture of oil-in-water systems for topical use. Likewise, [C_1_MIm]­[(MeO)_2_PO_2_] (Table 3, entry 2), used as the polar phase of IL-in-oil microemulsions, enabled acyclovir and methotrexate incorporation into 8–34 nm droplets with low polydispersity and no cytotoxicity in 3-D skin models.^58^

Oleophilic ILs such as [C_8_MIm]­[PF_6_] (Table 3, entry 3) supported sustained vemurafenib release and demonstrated short-term tolerability in ex vivo and rat-skin studies; however, the intrinsic cytotoxicity and hydrolytic instability of PF_6_^–^ ILs significantly limit their biomedical applicability.^59^ In contrast, choline geranate (CAGE) formulations circumvent these issues. In human studies, CAGE–caffeine gels improved dermal delivery without irritation (Table 3, entry 4), while CAGE–nobiletin systems increased oral bioavailability by ∼20-fold versus the crystalline drug, producing hypoglycemic effects in rats (Table 3, entry 5).^60,61^

Amino-acid-ester ILs such as [GlyC_1_]­[Cl] and [LeuC_12_]­[Cl] (Table 3, entry 6) further exemplify safe and potent permeation enhancement, increasing 5-FU flux by >7-fold compared with control formulations and outperforming Azone.^62^ Mechanistic evaluations demonstrated reversible disruption of stratum-corneum lipids and keratin organization—via lipid fluidization, selective extraction, and protein loosening—without irritation or loss of viability in vitro or in vivo.

IL-based hydrogels also serve as high-performance transdermal matrices. Self-assembling hydrogels formed from [Cho]­[Ala] or [Cho]­[Pro] with oleic acid (Table 3, entry 7) increased 5-FU solubility to >150 mg/mL and enhanced transdermal flux from 0.001 μM to 250 μM in PBS—an improvement exceeding 10^5^-fold relative to the initial medium—while maintaining healthy-cell viability.^63^

API-to-IL conversion provides an additional route to modulate the permeability. Ionic forms of metformin ([Met]­[DH], [Met]­[Doc], Table 3, entry 8) increased lipophilicity (log *P* ≈ 1.1 vs – 1.96 for MetHCl), resulting in 47-fold higher skin permeability and 53% in vivo bioavailability in rats, without irritation.^64^ Similarly, [Lev]­[Ole] (Table 3, entry 9) improved levocetirizine *C*_max_ and AUC by 4.6-fold and 5.4-fold, respectively, while being fully miscible with pharmaceutical oils and requiring no additional enhancers.^65^

Finally, IL gels derived from meglumine and dicarboxylic acids, such as [MgM]_1_ [Aza]_2_ (Table 3, entry 10), achieved 10% flurbiprofen solubilization and enhanced transdermal flux to 8.78 μg/cm^2^/h (3.2× vs control) without disrupting stratum-corneum lipids.^66^ Fourier transform infrared spectroscopy (FTIR) studies revealed selective interactions with keratin while preserving the lipid architecture, supporting safe and effective delivery of poorly soluble actives.

Collectively, entries 1–10 demonstrate that hydrophilic and amino-acid-derived ILs reproducibly enhance drug solubility (10^3^–10^5^×) and permeability (up to 7× vs controls) while maintaining cytocompatibility, whereas PF_6_^–^-based ILs remain limited by cytotoxicity and instability.

#### 3.3.2 Structural and Multifunctional Roles of ILs in Topical Systems

Beyond solubilization, ILs frequently act as structural and functional elements within soft materials, influencing rheology, interfacial assembly, drug loading, and release behavior. For instance, pressure-assisted 3-D printed films containing [Lid]­[Ibu] (Table 3, entry 11) supported amorphous drug incorporation and polymer-dependent release kinetics, enabling a synergistic analgesic/anti-inflammatory mucoadhesive platform.^67^ This system highlights how API-ILs can combine therapeutic activity with formulation versatility in oral mucosal applications.

ILs have also been incorporated into hydrogels and gel-emulsions, where they modulate viscosity, solubilize APIs, and provide intrinsic antimicrobial activity. The amphiphilic [C_6_Pyr]­[Cl] (Table 3, entry 12) reduced gel viscosity and doubled caffeine permeation across porcine skin relative to an IL-free control, while maintaining lower cytotoxicity than sodium dodecyl sulfate and preserving antimicrobial efficacy at MIC levels.^68^ Such multifunctionality—simultaneously acting as solvent, permeation promoter, and preservative—illustrates the formulation-integrating potential of ILs.

At the nanoscale, ILs function as interfacial stabilizers and self-assembly modulators. [C_4_MIm]­[Br] (Table 3, entry 13) increased silver-nanoparticle retention on skin by ∼1.7× without compromising mechanical flexibility or release kinetics.^69^ The choline–oleate IL [Cho]­[Ole] (Table 3, entry 14) formed stable micellar systems in IPM/Span-20 with >97% paclitaxel encapsulation and a 5-fold flux increase versus traditional surfactants.^70^ Salicylate ILs such as [C_2_MIm]­[Sal] and [C_16_Pyr]­[Sal] (Table 3, entry 15) exhibited low critical micelle concentration values (0.19 mM) and strong albumin binding (*K*_d_ < 100 μM), functioning as dual surfactant–API systems that combine delivery enhancement with salicylate pharmacology.^71^

In polymeric nanocarrier systems, choline-based ILs ([Cho]­[Cl], [Cho]­[OAc], [Cho]­[Buty]; Table 3, entry 16) tuned the micellization behavior of Pluronic carriers for curcumin delivery, reducing particle size and enhancing selectivity toward PC-3 prostate-cancer cells while preserving >90% HUVEC viability.^72^ IL-modified vesicular carriers further demonstrate structural control: Transfersomes containing [Cho]­[Gly] or [C_2_MIm]­[Gly] (Table 3, entry 17) exhibited smaller vesicle sizes (∼300 → <200 nm), ∼20% higher encapsulation efficiency, more negative zeta potential, and 90-day colloidal stability without cytotoxicity.^73^ These ILs improved lipid–drug interactions and reduced vesicle aggregation, contributing to more efficient transdermal transport.

Collectively, these findings show that ILs can govern interfacial behavior, nanostructure organization, and material rheology, enabling fine-tuning of particle dimensions, drug-carrier interactions, and release kinetics without compromising biocompatibility. Their multifunctional roles—spanning solvent, codrug, surfactant, rheology modifier, and interfacial stabilizer—underscore their value as structural building blocks in advanced topical and transdermal delivery systems.

#### 3.3.3 Advanced and Stimuli-Responsive Topical Platforms

The structural versatility of ILs has enabled advanced delivery devices capable of site-specific and on-demand release. Poly­(ionic-liquid) microneedles (Figure 7) (Table 3, entry 18) integrated NO-releasing imidazolium networks that penetrated the dermis painlessly and eradicated *C. albicans* biofilms, reduced inflammation, and promoted angiogenesis in infected mice relative to untreated controls.^11^

**Figure 7:**
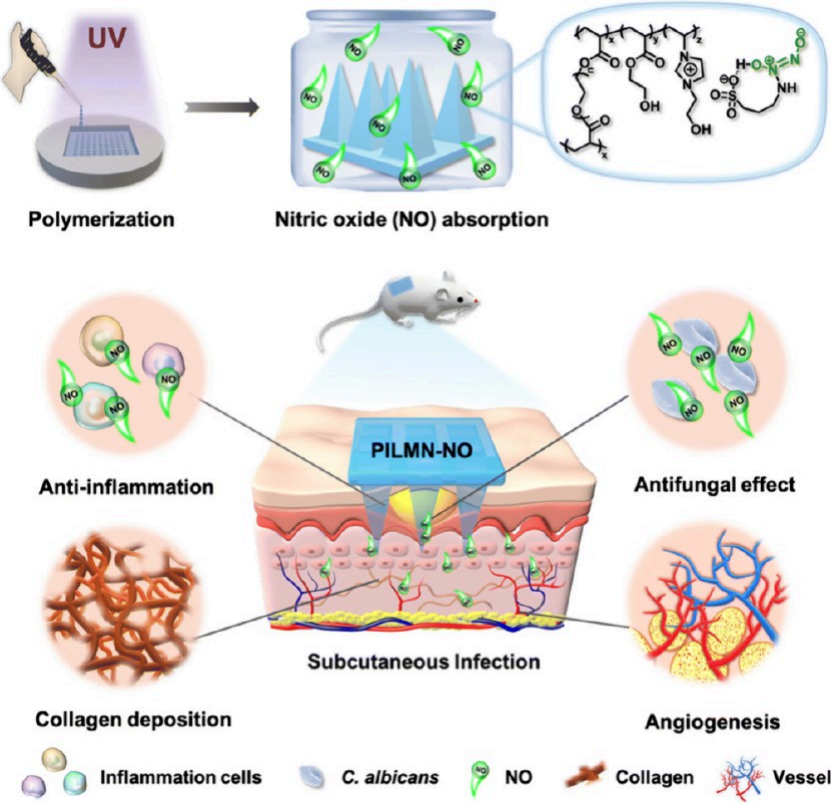
Schematic illustration of the synthesis of NO-releasing imidazolium-type PIL-based microneedle patches (PILMN-NO) and corresponding fungi killing, anti-inflammation, collagen deposition, and angiogenesis activities for promoting infection elimination and accelerating wound healing. Reprinted with permission from ref 11. Copyright 2023 Royal Society of Chemistry. Permission conveyed through the Copyright Clearance Center, Inc.

CAGE-agarose gels (Table 3, entries 19 and 20) permeated dense biofilms of *Porphyromonas gingivalis* and related periodontal pathogens, reduced alveolar bone loss, and lowered bacterial burden in ligature-induced periodontitis models, while maintaining safety after repeated applications.^74,75^ Mechanistic studies confirmed matrix penetration, extracellular-polymer disruption, and bactericidal activity without the development of resistance.

A pH- and temperature-responsive hydrogel based on [Cho]­[Ole] (Table 3, entry 21) formed worm-like micelles at elevated ionic strength, yielding a viscoelastic, skin-adherent 3D network.^76^ Gel collapse at pH 5.2 triggered rapid doxorubicin release, achieving ≈100-fold higher transdermal flux than aqueous doxorubicin (DOX) (63 μg/cm^2^; 7.4 μg/cm^2^ h), demonstrating how IL-structured matrices can couple passive diffusion with environmental responsiveness.

Further control over spatiotemporal delivery was achieved with photoresponsive azobenzene imidazolium salts (Table 3, entry 22) embedded within montmorillonite layers.^77^ UV irradiation expanded the clay galleries, releasing ≈80% PABA in 8 h compared with 30% in the dark, exemplifying light-activated molecular actuation.

Across these systems, ILs evolve from simple solvents to architectural and actuating components, enabling programmable drug distribution, biofilm permeation, environmental triggering, and localized therapeutic action. Collectively, they broaden the design space of topical therapy by merging structural function, intrinsic bioactivity, and stimuli-responsive behavior.

#### 3.3.4 Targeted Therapeutic Applications: Anti-Inflammatories, Antimicrobials, Biologics, and Vaccines

##### 3.3.4.1 Anti-Inflammatories and Analgesics

The conversion of APIs into ILs has broadened therapeutic options for topical anti-inflammatory and analgesic therapies by coupling improved biopharmaceutical properties with reduced skin toxicity. Amino-acid–salicylate ILs such as [ValC_3_]­[Sac] and [PheC_1_]­[Sac] (Table 3, entry 23) tripled aqueous solubility relative to salicylic acid and increased thermal stability, while elevating IC_50_ values in 3T3 and HaCaT cells.^78^ At noncytotoxic concentrations, they normalized IL-6 expression, indicating genuine anti-inflammatory activity in addition to permeation enhancement.

Hydrogen-bonded [Pip]­[Ket] (Table 3, entry 24) raised ketoprofen solubility by 85% and doubled dermal permeation versus the physical mixture, leading to reduced paw-edema AUC and enhanced COX-2/15-LOX inhibition in vivo.^79^ Similarly, [ProC_2_]­[Ibu] (Table 3, entry 25) increased ibuprofen flux ≈10-fold versus saturated solution while maintaining low fibroblast toxicity.^80^ Clinical translation of this concept was illustrated by the ILTS ointment (Table 3, entry 26), in which an IL matrix delivered STAT6-decoy ODNs ≈10-fold more efficiently than petrolatum, markedly suppressing IL-4 and IL-13 in dermatitis models.^81^

API–API designs further enable rational codelivery. [Lid]­[Edo] and [Lid]­[Dic] (Table 3, entries 27–28) increased etodolac solubility 96×, improved transdermal flux ≈9×, and afforded sustained dual-drug release for >12 h from cryogel patches, with good stability.^82,83^ In contrast, the neutral eutectic Lid·Ibu (Table 3, entry 29) showed AUC_0_-_4h_ ≈ 1763 μM h versus 12 μM h for [Lid]­[Doc], highlighting how ionization state dictates systemic exposure.^84^ Proline–naproxen salts [ProiC_3_]­[NAP] and [ProC_3_]­[NAP] (Table 3, entry 30) increased dermal delivery ≈3.8-fold vs free naproxen, with >80% L929 viability at 2 mM.^85^ Collectively, these systems show that tuning IL ion pairs can simultaneously optimize solubility, flux, and anti-inflammatory efficacy while preserving skin compatibility.

##### 3.3.4.2 Antimicrobials and Antifungals

For infectious dermatoses, ILs support both the stabilization of antimicrobial agents and their penetration into infected niches. CAGE–bacteriophage hydrogels (Table 3, entry 31) maintained phage viability, promoted diffusion through skin relative to hydroxyethyl cellulose gels, and showed no cytotoxicity or genotoxicity, enabling selective targeting of Staphylococcus intermedius.^86^ The cholinium–citrate IL [Cho]_3_[Cit]_1_ (Figure 8) (Table 3, entry 32) increased 5-ALA flux and intradermal accumulation up to 10× vs aqueous 5-ALA, reducing Propionibacterium acnes burden and IL-1β levels in rats without barrier damage, supporting its use as a pretreatment for photodynamic therapy.^87^

**Figure 8:**
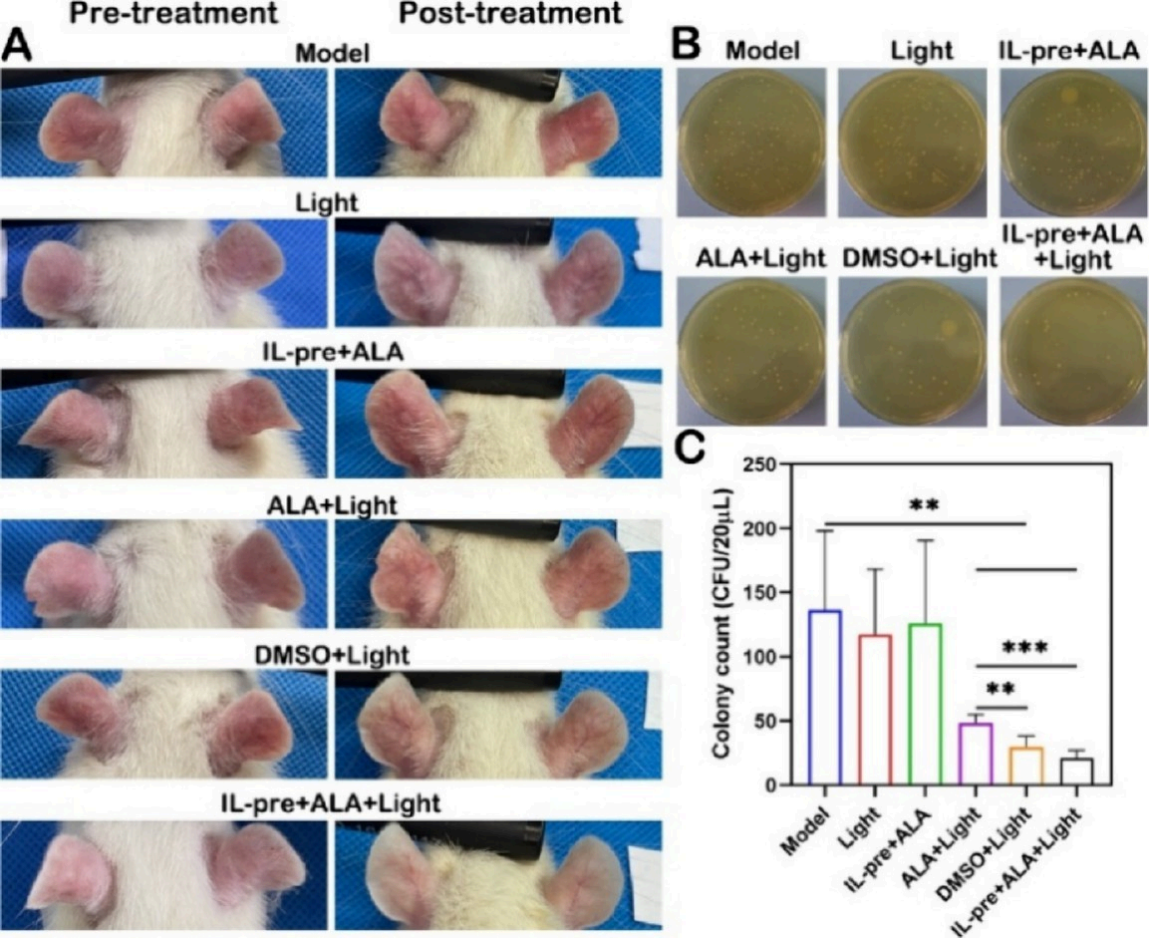
In vivo antiacne effects. (A) Representative images showing the apparent changes in the pre- and post-treated ears in *P. acnes*-induced rats. Photographic images (B) and counting statistics (C) of in vivo bacterial colonies spread on a BHI plat. Reproduced with permission from ref 87. Copyright 2024 American Chemical Society.

Imidazolium IL–based emulsions ([C_12_MIm]­[Br], [C_16_MIm]­[Br]; Table 3, entry 33) strengthened drug–IL interactions and organized micellar structures, yielding IC_50_ values against *Candida* spp. up to 1000× lower than fluconazole alone while remaining nonirritant in HET-CAM assays.^88^ Ternary [Cho]­[Gly]/EtOH/IPM systems (Table 3, entry 34) achieved acyclovir delivery of ≈58 μg cm^–2^ in 24 h—substantially higher than controls—through reversible disruption of stratum-corneum lipid packing confirmed by FTIR, yet retained good epidermal tolerability.^89^ These examples underscore how ILs can be engineered to balance strong antimicrobial performance with acceptable local safety.

##### 3.3.4.3 Biologics, Peptides, and siRNA

The barrier-modulating yet biocompatible nature of many ILs is increasingly being exploited for macromolecular therapeutics. A composite [Cho]_1_[Hyd]_2_/[Cho]_2_[Get]_3_ formulation (Table 3, entry 35) protected siRNA, enhanced uptake in keratinocytes and porcine skin, and attenuated psoriatic lesions and PASI scores in mice without local or systemic toxicity.^90^ Solid-in-oil nanoemulsions incorporating [C_12_MIm]­[NTf_2_] (Table 3, entry 36) enabled dermal delivery of 45 kDa ovalbumin and elicited systemic IgG responses in the absence of conventional adjuvants.^91^

For polysaccharides, [Cho]­[Mal] (Table 3, entry 37) enhanced dextran penetration through epidermis and dermis compared with saline, driven by transient lipid extraction while maintaining >95% HEK-A viability and irritation-free skin in vivo.^92^ Bacterial-nanocellulose/xanthan films containing insulin and [Cho]_1_[Ger]_2_ (Table 3, entry 38) preserved protein structure and achieved transdermal delivery up to 1.05 μg mm^–2^—far exceeding free insulin—without cytotoxic or genotoxic effects.^93^ Together, entries 35–38 demonstrate that IL-based matrices can safely deliver nucleic acids, proteins, and polysaccharides by coupling barrier modulation with structural protection.

##### 3.3.4.4 Vaccines and Transcutaneous Immunotherapy

IL-containing systems have also emerged as noninvasive vaccine and cancer-immunotherapy platforms. [C_4_MIm]­[Br] microemulsions (Table 3, entry 39) increased 5-FU solubility from 12.2 to 31.2 mg mL^–1^ and achieved ≈91% cutaneous permeation (≈50 μg h^–1^ cm^–2^, 24 h), outperforming commercial cream and driving complete regression of DMBA/TPA-induced skin tumors in mice without irritation.^94^ Although they are primarily cytotoxic therapies, these data highlight the potential of IL-based microemulsions for high local drug loads.

For antigen delivery, [Cho]­[Ole] (Table 3, entry 40) enabled solubilization and dermal transport of the SIINFEKL peptide in EtOH/IPM vehicles, increasing epidermal and dermal deposition, CD8^+^ T-cell infiltration, and tumor suppression versus vehicle alone, with good safety in 3D human skin models and murine histology.^95^ [TEtOHA]­[Lact] (Table 3, entry 41) facilitated sequential transcutaneous delivery of the adjuvant R848 followed by WT1 peptide, producing strong CTL responses and tumor control in murine models without dermal toxicity.^96^ IL-based vaccination strategies therefore exploit both their solubilizing capacity and their ability to modulate skin permeability and local immune responses.

##### 3.3.4.5 Synthesis and Perspectives

Across entries 23–41, ILs span a continuum from “passive” permeation enhancers to active structural and pharmacological components. By rationally selecting ion pairs and embedding them in appropriate soft matrices, these systems deliver consistent gains in solubility, transdermal flux, local bioavailability, and therapeutic outcome while preserving skin integrity. Compared with oral or injectable routes—which prioritize systemic exposure and strict hemocompatibility—topical IL formulations emphasize interfacial engineering and localized pharmacological action, generally limiting the systemic burden. Nonetheless, systematic long-term safety and sensitization studies will be essential before widespread clinical deployment of IL-based dermal therapeutics and vaccines.

### 3.4 Intranasal and Nose-to-Brain Delivery Using Ionic Liquids and Ionogels

The intranasal route has gained substantial traction as a platform for applying ILs and ionogels in brain-targeted pharmaceutical delivery. ILs can simultaneously act as molecular solubilizers, barrier modulators, and biocompatible matrices, enabling the dissolution enhancement, mucus penetration, and controlled tight-junction modulation. Unlike oral or topical formulations, nasal systems must meet stringent criteria related to mucociliary compatibility, viscosity–retention balance, and rapid, yet reversible, epithelial modulation. Recent advances (Table 4) demonstrate how IL-based systems integrate these functions to achieve efficient N2B transport of both small molecules and macromolecular therapeutics.

**Table 4:**
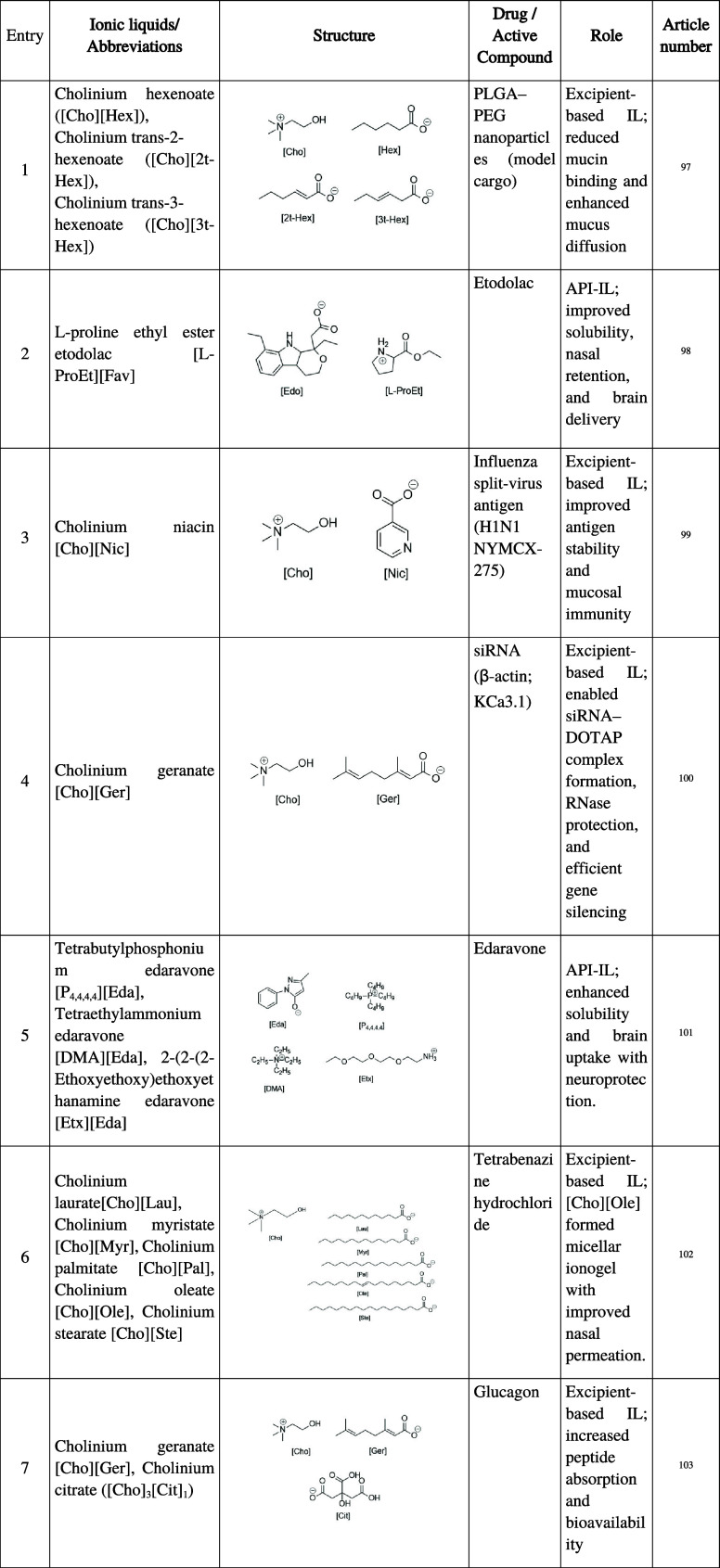
Summary of Ionic Liquids Employed in N2B Pharmaceutical Formulations, Including Abbreviations, Chemical Structure, Active Compound, Functional Role, and Corresponding Reference Numbers

#### 3.4.1 IL-Mediated Mucus Penetration and Enhanced Transport of Small Molecules

At the nanoscale, ILs regulate interactions among drugs, mucus, and epithelial membranes, improving the diffusional access to the olfactory and trigeminal pathways. PLGA–PEG nanoparticles coated with choline carboxylate ILs (Table 4, entry 1) weakened nanoparticle–mucin binding and partially unfolded mucin secondary structures.^97^ This restructuring increased mucus diffusivity without cytotoxicity, enabling more efficient transport toward the olfactory epithelium.

API–IL, composed of etodolac and l-proline ethyl ester ([ProOEt]­[ETD]) were developed for N2B delivery (Table 4, entry 2).^98^ Compared with aqueous etodolac, the IL enhanced solubility by ∼480-fold and produced 7-fold higher brain exposure in mice due to prolonged mucosal residence and increased viscosity. This allowed effective inhibition of COX-2 with no signs of irritation. These examples illustrate how IL chemistry can simultaneously improve dissolution, mucosal retention, and epithelial penetration for small-molecule CNS therapeutics.

#### 3.4.2 Delivery of Biologics, Peptides, and Nucleic Acids via IL-Based Systems

ILs are also powerful tools for stabilizing fragile biomolecules and enabling macromolecular transport through the nasal mucosa. Oil-in-ionic-liquid nanoemulsions were designed using choline nicotinate ([Cho]­[Nic]) with squalene and Tween 80 that functioned simultaneously as a solubilizing medium, stabilizer, and intrinsic immunoadjuvant (Table 4, entry 3).^99^ The optimized formulation (≈169 nm, PdI 0.07) remained stable for 12 months at 25 °C and increased mucosal sIgA secretion by ∼25-fold relative to antigen solution, outperforming the MF59 benchmark.

Complementarily, siRNA was formulated with DOTAP in [Cho]­[Ger], producing cationic lipid nanocomplexes that protected siRNA from RNase degradation for >4 h and maintained >40% nasal retention at 12 h.^100^ These complexes achieved >50% β-actin mRNA knockdown and significantly reduced IgE, PGD_2_, and histamine in allergic-rhinitis rats while restoring ZO-1 expression. Together, these findings highlight ILs as versatile components for stabilizing biomacromolecules, enhancing cellular uptake, and modulating nasal immune responses.

#### 3.4.3 API–ILs and Ionogel Architectures Enabling Brain Targeting and Controlled Release

Recent work extends beyond excipient-based IL systems to API–ILs and advanced IL-containing matrices. An edaravone-based API–IL was synthesized by pairing the edaravone anion with an ethoxy cation.^101^ The IL displayed a 5.9-fold higher aqueous solubility than crystalline edaravone and spontaneously formed ∼182 nm nanocomposites upon hydration. Intranasal administration produced 3.4-fold higher brain levels, reducing the infarct volume and cerebral edema in ischemic rats without toxicity.

Ionogels further expand the structural possibilities for nasal delivery. [Cho]­[Ole] micelles were embedded within Pluronic F-127 to create a thermoreversible gel for tetrabenazine hydrochloride (TBZ) (Table 4, entry 6).^102^ The optimized ionogel exhibited 38% drug loading, 83% entrapment efficiency, and gelation at ∼33 °C, achieving 1.37-fold higher cumulative release and 1.42-fold higher ex vivo permeation vs Pluronic gel alone. The formulation remained stable for 90 days at 4 °C and followed zero-order kinetics.

Glucagon formulations based on [Cho]­[Ger] and [Cho]­[Cit] transiently modulated tight junctions by reorganizing ZO-1 and occludin, with complete recovery within hours (Table 4, entry 7).^103^ Nasal administration achieved relative bioavailabilities of 99 and 86%, comparable to subcutaneous injection, with rapid glycemic recovery and no airway irritation. These results confirm IL-based ionogels and API–ILs as promising vehicles for on-demand CNS targeting and controlled peptide release.

#### 3.4.4 Mechanistic Themes and Translational Outlook

Across these studies, three recurring mechanistic themes emerge: (i) Molecular-level solubilization—ILs greatly enhance solubility of poorly soluble small molecules and API–IL systems, often by >100-fold; (ii) Mucus/epithelium modulation with reversible effects—ILs reduce mucin binding, restructure glycoproteins, modulate tight junctions, and increase epithelial permeability—yet typically allow full recovery; and (iii) Structuring and stabilization of complex formulations—ILs enable nanoemulsions, nanocomposites, micellar ionogels, and cationic lipid complexes with long-term stability and preserved biomolecular integrity.

Collectively, these properties provide an integrated solution to the longstanding limitations of nasal drug delivery—short residence time, limited permeability, and poor stability of biologics—positioning ILs and IL-based ionogels as versatile platforms for noninvasive, brain-targeted, and macromolecule-capable nasal therapeutics.

### 3.5 Ocular Formulations

Ocular drug delivery poses distinct challenges due to the eye’s multiple protective barriers, rapid tear turnover, and the demand for localized therapy with minimal systemic absorption. Conventional eye drops frequently suffer from low bioavailability, instability of labile drugs, and patient nonadherence. ILs have recently emerged as multifunctional agents capable of improving solubility, permeability, and formulation stability simultaneously, thereby enhancing the therapeutic index of ophthalmic systems (Table 5).

**Table 5:**
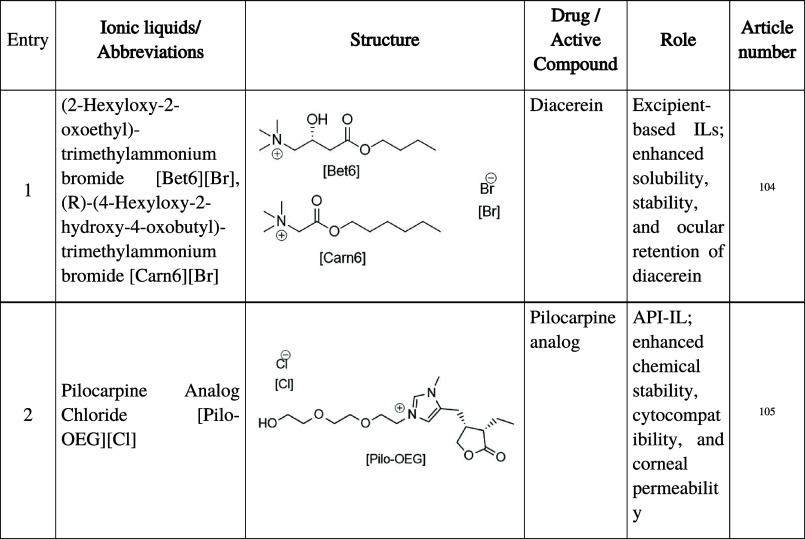
Summary of Ionic Liquids Employed in Ocular Pharmaceutical Formulations, Including Abbreviations, Chemical Structure, Active Compound, Functional Role, and Corresponding Reference Numbers

#### 3.5.1 ILs as Solubilizers and Stability Enhancers for Ophthalmic Drugs

Betaine and L-carnitine derivatives ([Bet_6_]­[Br] and [Carn_6_]­[Br]; Table 5, entry 1) were incorporated into eye drop formulations containing diacerein (DIA), a poorly soluble and hydrolysis-prone anti-inflammatory drug.^104^ These ILs increased the DIA solubility to 0.34 M and stabilized the drug above their critical aggregation concentration (0.27 M), with [Bet_6_] preserving up to 85% of DIA after 6 h. The formation of nanoaggregates imparted mucoadhesive protection, extending residence time on the ocular surface.

In vitro assays demonstrated high cytocompatibility (IC_50_ ≥ 7.5 mg/mL in BALB/3T3 fibroblasts) and broad antimicrobial activity against *Staphylococcus aureus* and *Pseudomonas aeruginosa*. Moreover, rhein—the active metabolite of DIA—suppressed *S. aureus* biofilm formation, reinforcing the antimicrobial contribution of the IL system.

In vivo studies in rabbits confirmed ocular tolerability with only mild and transient conjunctival irritation. Compared to conventional DIA suspensions, IL-based eye drops achieved ≥2.5-fold higher mean residence time (MRT) and ≥3-fold higher AUC, particularly with [Bet_6_].

#### 3.5.2 API–ILs for Enhanced Corneal Permeation and Therapeutic Performance

In another approach, [Pilo–OEG]­[Cl] was developed, an ionic liquid analog of pilocarpine obtained through reaction with a chlorinated oligoethylene glycol derivative (Table 5, entry 2).^105^ This compound exhibited enhanced chemical stability, maintained physiological pH (7.3), and prevented hydrolytic degradation into isopilocarpine.

[Pilo–OEG]­[Cl] also showed excellent cytocompatibility in human corneal epithelial cells (HCE-2) up to 50 mM, no hemolytic activity, and cytokine (IL-6, TNF-α) expression comparable to that of pilocarpine hydrochloride (PiloHCl). Importantly, the IL achieved a 6-fold increase in corneal permeability (1.3 × 10^–5^ cm/s) relative to PiloHCl, attributable to its predominantly nonionized form (∼94%) at physiological tear pH (∼7.2), which favored passive diffusion.

In vivo studies in normotensive Brown Norway rats demonstrated a more sustained reduction of intraocular pressure following two administrations compared with PiloHCl. Histological analysis confirmed the absence of inflammation or tissue damage, aside from minor epithelial exfoliation considered physiologically normal.

#### 3.5.3 Remaining Challenges and Future Directions

Collectively, these studies demonstrate that ILs can significantly enhance ophthalmic formulations by increasing solubility, chemical stability, antimicrobial activity, corneal permeability, and ocular residence time—addressing key limitations of conventional eye drops.

However, despite promising preclinical outcomes, long-term ocular safety, regulatory classification, and manufacturing reproducibility of IL-based systems remain to be fully established. Additional translational research, including chronic toxicity assessments, mechanistic studies on tear-film interactions, and controlled clinical trials, will be essential to consolidating the role of ILs in next-generation ophthalmic therapeutics.

### 3.6 Advanced Drug Release Formulations

Recent advances in IL design have expanded their role beyond solubilization and stabilization toward the engineering of drug delivery systems with tunable release kinetics, targeted functionality, and responsiveness to external or physiological stimuli. These properties have enabled ILs to function as structural components, modulators of molecular transport, or dynamic actuators within soft and implantable materials. Table 6 compiles representative IL-based hydrogels, nanocomposites, ionogels, implantable depots, and biopharmaceutical constructs that illustrate these emerging strategies.

**Table 6:**
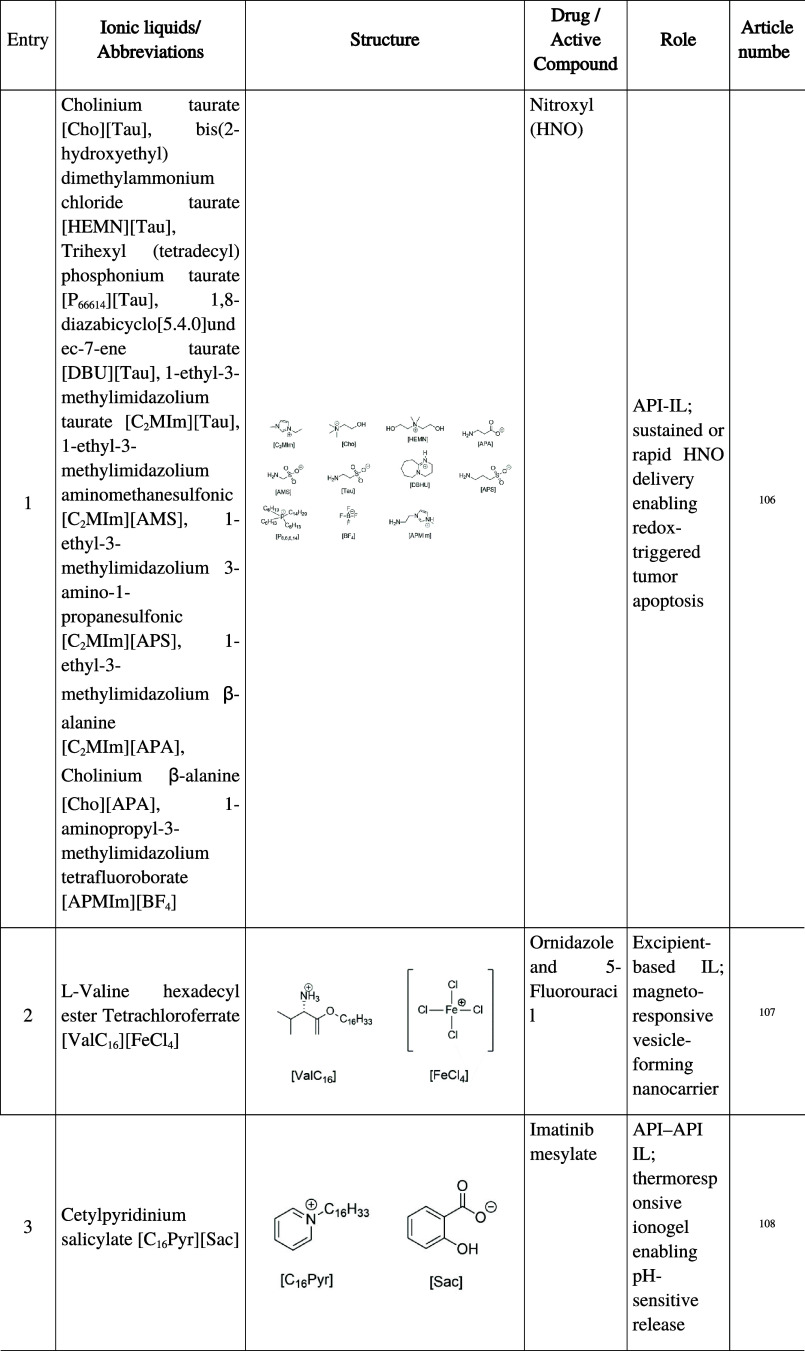

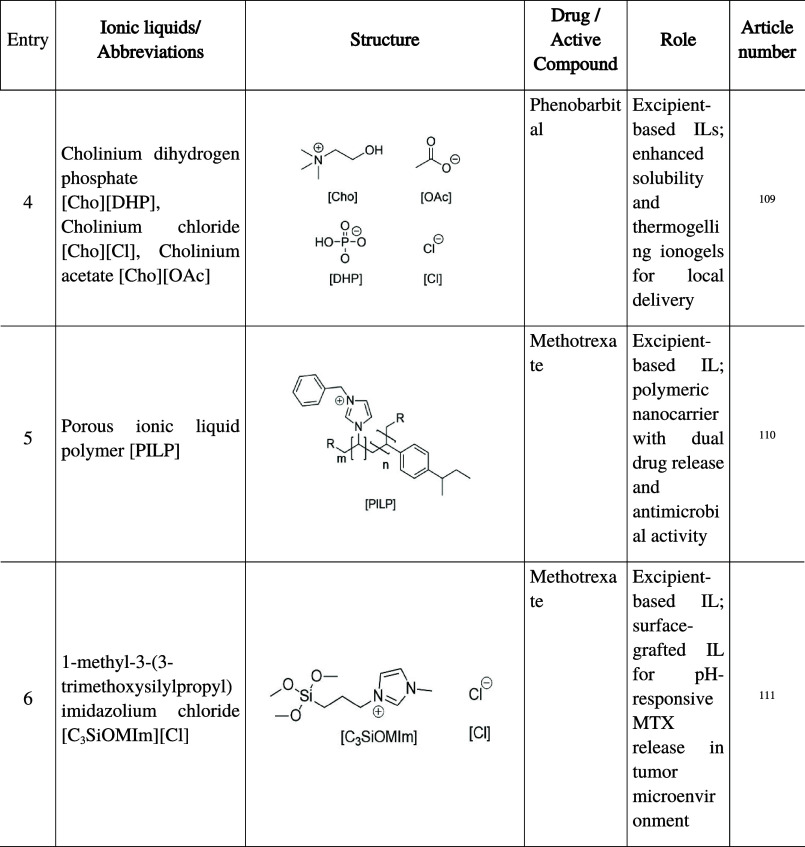

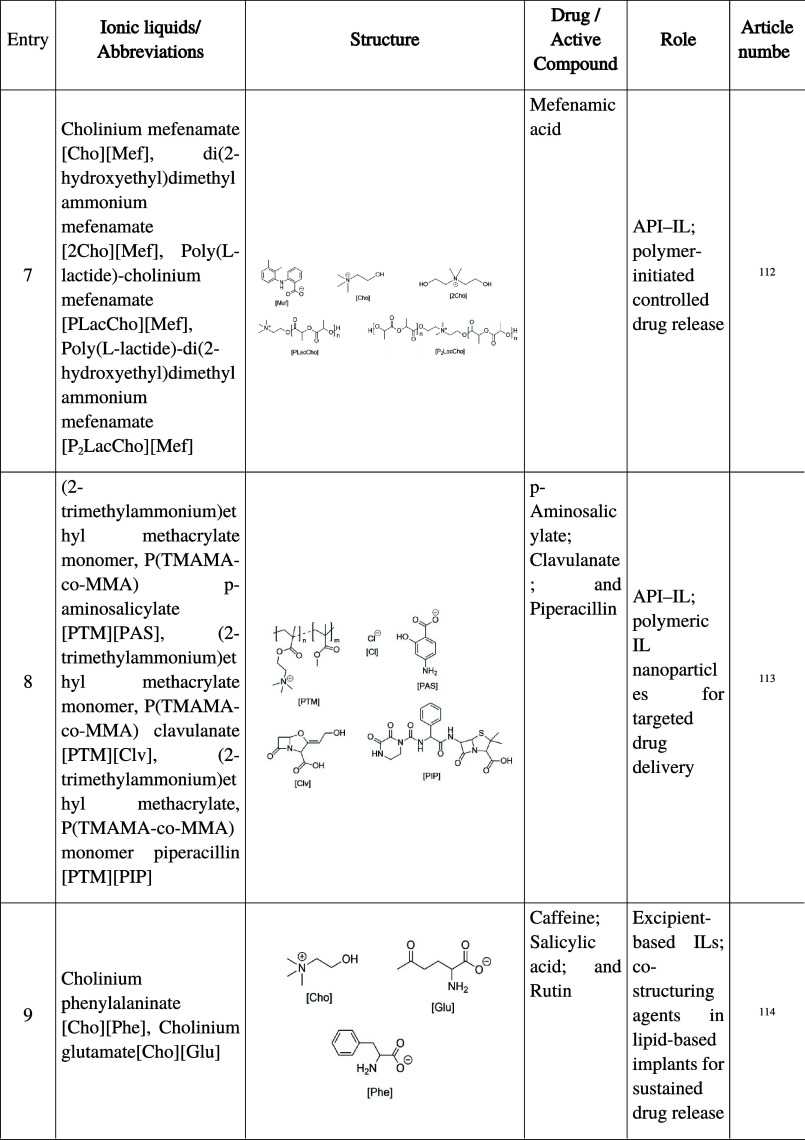

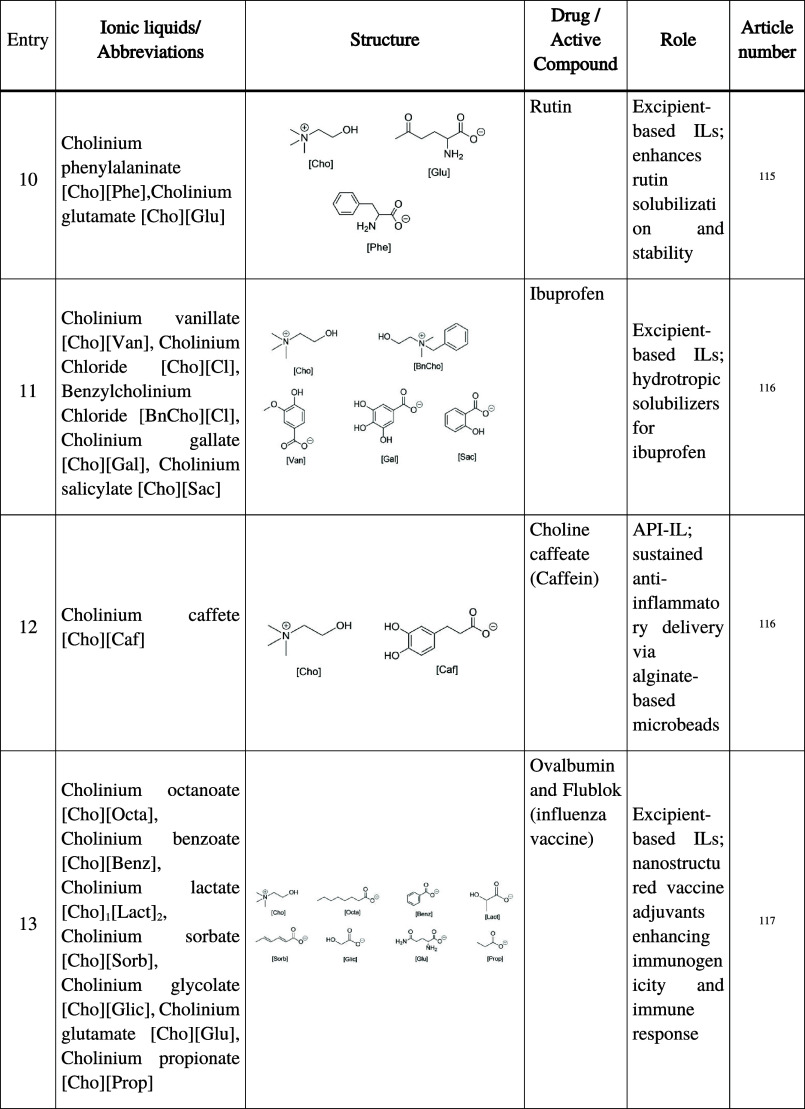

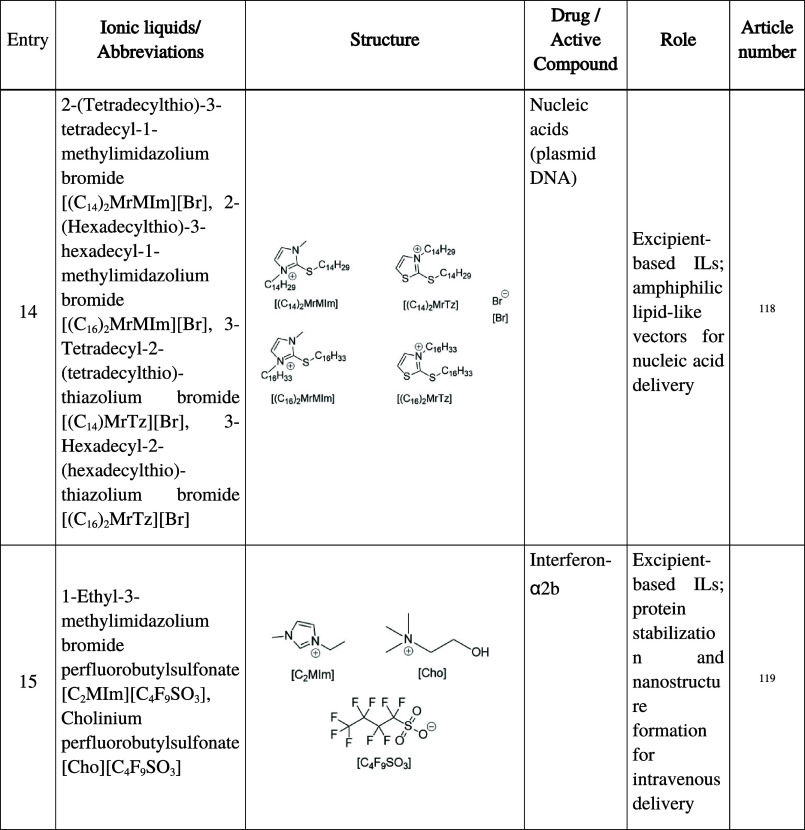
Summary of Ionic Liquids Employed in Advanced Drug Release Formulations, Including Abbreviations, Chemical Structure, Active Compound, Functional Role, and Corresponding Reference Numbers

#### 3.6.1 Gasotransmitter-Generating ILs (Redox-Modulated Therapy)

Ionic liquids can also be engineered as bioactive donors, releasing therapeutic gases directly at the target site. Lv et al. developed a family of IL–NONOates (Table 6, entry 1) that generate nitroxyl (HNO) through finely tuned intramolecular hydrogen bonding between the NONOate moiety and functional groups within the anion (Figure 9).^106^ Molecular design dictated release kinetics: [Cho]­[APA]–NONOate exhibited prolonged HNO release (*t*_1/2_ = 1061 min), whereas [APMIm]­[BF_4_]–NONOate, which lacks stabilizing interactions, released HNO rapidly (*t*_1/2_ = 4.2 min).

**Figure 9:**
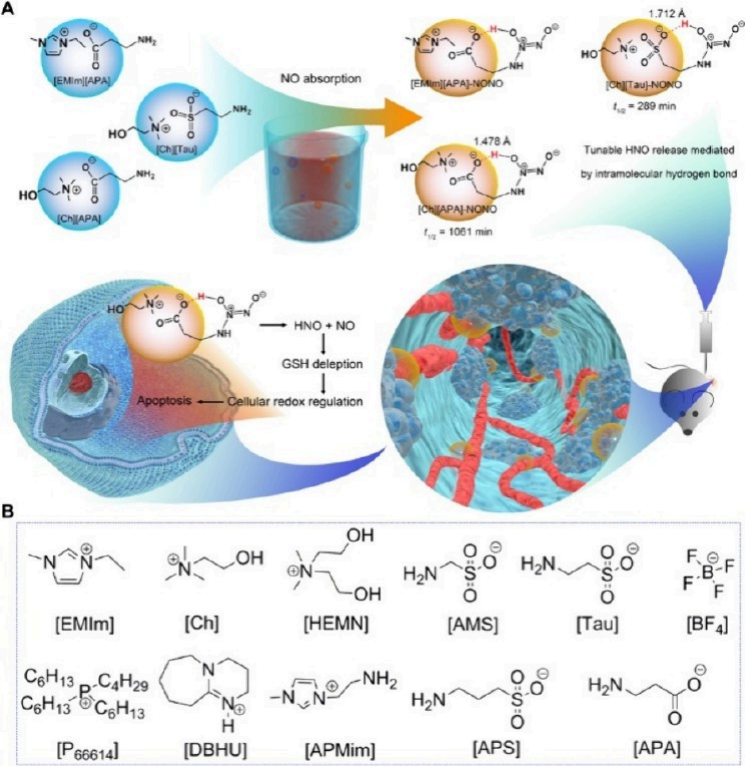
(A) Schematic illustration of mechanisms for tunable release of HNO and tumor inhibition by IL-NONOates mediated by an intramolecular hydrogen bond. (B) Structures of cations and anions used in this work. GSH, glutathione. Reproduced with permission from ref 106. Copyright 2023 Elsevier.

In vitro, these IL-NONOates elevated reactive nitrogen species (RNS), depleted intracellular glutathione, and induced apoptosis in CT-26 and MCF-7 tumor cells. In vivo, subcutaneous administration of [Cho]­[APA]–NONOate in BALB/c mice bearing CT-26 colorectal tumors produced a ≈ 55% reduction in tumor volume over 22 days and a 5.6-fold increase in intratumoral RNS relative to controls.

These results demonstrate how precise molecular control over the NONOate architecture allows ILs to couple redox modulation with localized antitumor activity, establishing IL-based gasotransmitter donors as a promising class of redox-responsive therapeutics.

#### 3.6.2 Stimuli-Responsive Hydrogels and Ionogels

Embedding ILs within hydrogel and ionogel matrices enables drug delivery systems that respond to external or physiological stimuli. Magneto-responsive hydrogels incorporating the paramagnetic surfactant [ValC_16_]­[FeCl_4_] (Table 6, entry 2) encapsulated ornidazole and 5-FU with efficiencies of ∼70–80%, releasing both drugs over 96 h according to the Korsmeyer–Peppas model.^107^ The IL strengthened the hydrogel architecture through vesicle-like structuring and ensured homogeneous distribution of the [FeCl_4_] anion, conferring robust magnetic responsiveness suitable for externally triggered drug release.

Thermoresponsive ionogels formed from [C_16_Pyr]­[Sal] (Table 6, entry 3) self-assembled into viscoelastic matrices capable of loading high amounts of imatinib mesylate.^108^ These gels exhibited reversible gel–sol transitions near 25 °C and pH-dependent release reaching ∼94% at pH 5, following Higuchi kinetics. Structural analyses revealed temperature-induced rearrangement from fibrous networks to cylindrical micelles, while surface erosion at the gel–fluid interface dominated release under acidic conditions—an arrangement well aligned with tumor-like microenvironments.

Complementary systems based on silk fibroin and choline-derived ILs ([Cho]­[OAc], [Cho]­[DHP], [Cho]­[Cl]; Table 6, entry 4) further demonstrated how anion selection tunes mechanical integrity, gelation temperature (35–49 °C), and drug solubilization capacity.^109^ In particular, [Cho]­[OAc] and [Cho]­[DHP] produced stronger, faster-forming gels than [Cho]­[Cl], emphasizing how IL-specific hydrogen-bonding patterns define the responsiveness and performance of ionogels.

Together, these examples highlight how rational IL selection—modulating hydrophobicity, hydrogen bonding, and counterion properties—enables dual or multistimuli responsive gel platforms capable of sustained drug release under tumor- or inflammation-relevant conditions.

#### 3.6.3 Polymeric Nanocarriers with Integrated Ionic Functionality

Integrating IL moieties into polymeric carriers yields adaptive nanostructures with enhanced encapsulation efficiency, environmental responsiveness, and inherent bioactivity. Porous ionic-liquid polymers doped with silver nanoparticles (Ag-PILP; Table 6, entry 5) enabled sustained methotrexate release (≈96% over 120 h) and exhibited antimicrobial activity against *S. aureus* and *E. coli* (MIC = 256 μg/mL) while maintaining fibroblast compatibility.^110^ Their mesoporous architecture, derived from 1-vinylimidazole and benzyl bromide, provided high drug-loading capacity and structural stability, supporting dual therapeutic roles where infection control and chemotherapy must be addressed simultaneously.

pH-responsive hybrid systems have also been developed by grafting polymethacrylic acid onto [C_3_SiOMIm]­[Cl]-modified silica nanoparticles (Table 6, entry 6).^111^ These constructs demonstrated complete methotrexate release under acidic conditions (pH 4), but only ≈30% at physiological pH 7.4 over 400 h, confirming their suitability for tumor-microenvironment–selective delivery. Cytotoxicity tests retained the antitumor activity of methotrexate against MCF-7 cells, supporting the use of IL-modified nanosilicas as environmentally selective platforms.

Pharmaceutical IL–polymer conjugates provide an additional strategy, exemplified by mefenamic-acid–based API–IL nanoparticles (Table 6, entry 7), which achieved encapsulation efficiencies of 72–94% and biphasic release kinetics modeled by pseudo-second-order behavior.^112^ Release performance was tunable via polymer molecular weight, dispersity, and hydrophilic–hydrophobic domain arrangement, highlighting the architectural control afforded by IL incorporation.

Finally, amphiphilic copolymers bearing quaternary-ammonium IL units ([PTM], Table 6, entry 8) have been engineered to deliver anionic antibiotics such as *p*-aminosalicylate, clavulanate, and piperacillin.^113^ These nanocarriers (≈170–290 nm) achieved drug-loading efficiencies of 43–76% and released up to 33% of the payload within 4 h. In vitro, they displayed selective cytotoxicity toward lung cancer cell lines (A549, H1299) over healthy bronchial epithelial cells (BEAS-2B), accompanied by apoptosis induction and modulation of IL-6/IL-8 expression. These effects suggest a dual antimicrobial and immunomodulatory role.

Altogether, these systems illustrate how ionic self-assembly, tailored counterions, and dynamic hydrogen-bonding interactions can convert conventional polymeric carriers into environmentally responsive nanoplatforms with synergistic therapeutic functions.

#### 3.6.4 Choline-Based Long-Acting and Implantable Systems

Choline-based ILs have proven to be versatile as biocompatible structuring agents in long-acting and localized formulations. [Cho]­[Phe] and [Cho]­[Glu] (Table 6, entry 9) were incorporated into lipid implants sustaining the release of caffeine, salicylic acid, and rutin for up to 140 days,^114^ while analogous PLGA multiple emulsions (Table 6, entry 10) achieved rutin encapsulation efficiencies of up to 72% with excellent colloidal stability.^115^ Despite their distinct dosage forms, both platforms illustrate the ability of choline ILs to maintain matrix integrity and support the prolonged delivery of lipophilic APIs.

Hydrotropic ILs such as [Cho]­[Van], [Cho]­[Gal], and [Cho]­[Sac] (Table 6, entry 11) significantly enhanced the aqueous solubility of ibuprofen—exceeding a 6000-fold increase relative to water—and outperformed traditional hydrotropes such as sodium toluenesulfonate and urea.^116^ These choline-derived ILs form nonmicellar aggregation domains, generating effective solubilization microenvironments while maintaining low cytotoxicity and intrinsic bioactivity, reinforcing their suitability as aqueous solubility enhancers.

In a bioinspired localized-therapy platform, [Cho]­[Caf] was encapsulated within alginate–acemannan microbeads for intra-articular delivery (Table 6, entry 12), yielding strong swelling behavior in PBS and sustained IL release over 7 days (Figure 10).^120^ The system supported ATDC5 cell viability and glycosaminoglycan production, and THP-1-derived macrophages exhibited reduced secretion of TNF-α and IL-6, confirming an anti-inflammatory effect of the phenolic IL within the natural-polymer matrix.

**Figure 10:**
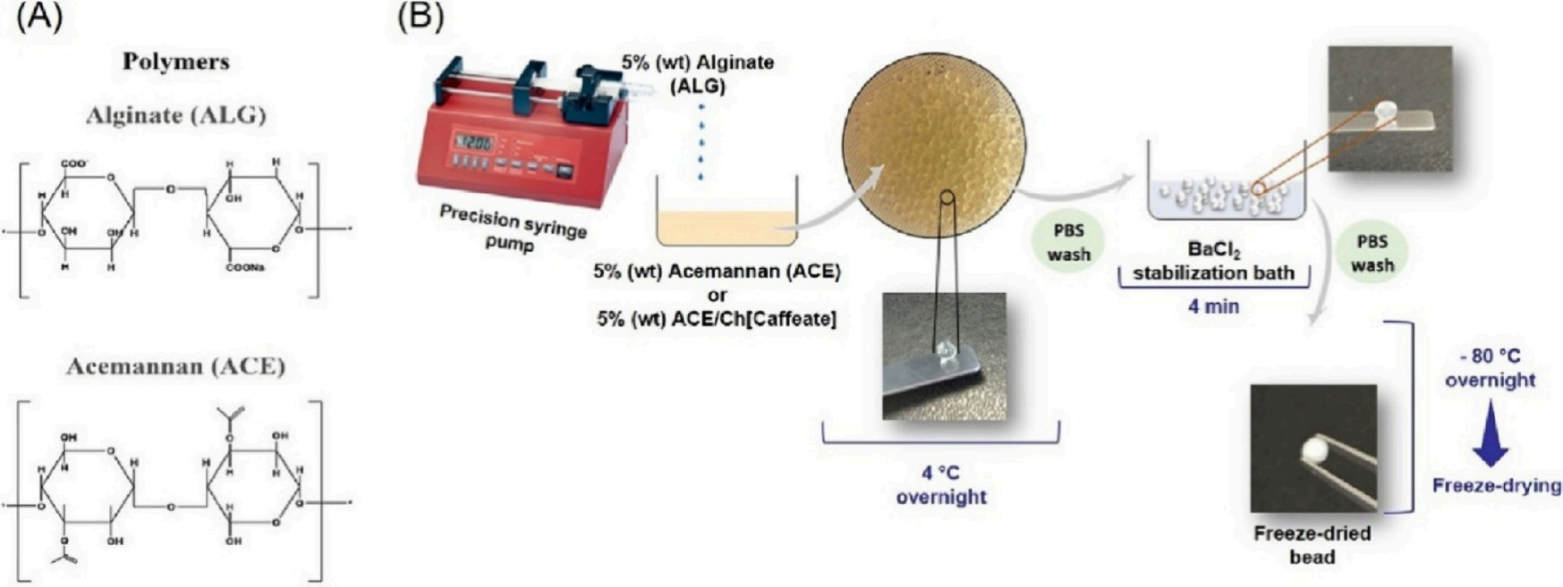
(A) Alginate and acemannan chemical structure. (B) Schematic representation for the preparation of the ALA and ALAC-based beads. Reproduced from ref 120. Copyright 2023 Elsevier B.V.

Collectively, these systems demonstrate that choline-derived ILs can reinforce structural stability, prolong drug release, and—when pharmacologically active—contribute to the therapeutic effect, positioning them as multifunctional components for long-acting and implantable delivery platforms.

#### 3.6.5 Ionic Liquids in Vaccines, Biologics, and Gene Therapy

ILs increasingly serve as structural stabilizers and immunomodulatory enhancers in biologic formulations. Choline sorbate ([Cho]­[Sorb]; Table 6, entry 13) self-assembled into ≈21 nm nanostructures that improved the stability and immunogenicity of ovalbumen and Flublok vaccines, promoting balanced Th1/Th2 immunity (IgG2c/IgG1 ≈ 1) and robust memory T-cell expansion through ferroptosis-driven dendritic-cell activation.^117^ Lipid-like ILs (LILs), exemplified by [(C_16_)_2_MrTz]­[Br] (Table 6, entry 14), mimicked lamellar cationic lipids and enabled ≈31% gene transfection in HEK 293T cells at reduced cytotoxicity compared with Lipofectamine 3000.^118^ Complementarily, fluorinated ILs ([C_2_MIm]­[C_4_F_9_SO_3_] and [Cho]­[C_4_F_9_SO_3_] Table 6, entry 15) formed lamellar aggregates that stabilized interferon-α_2_b in biological fluids without perturbing its secondary structure.^119^

These case studies highlight the capacity of ILs to couple physicochemical versatility with biological function. Ionic self-assembly, dynamic hydrogen bonding, and reversible interfacial reorganization commonly underpin their release behavior, which can span hours to months depending on the formulation architecture. Injectable and implantable IL systems require carefully tuned isotonicity and biodegradability, while topical and intranasal systems benefit from transient and reversible barrier modulation. Overall, the formulations in Table 6 exemplify how rational IL design converts them from inert solvents into active structural components that enable targeted delivery and advanced biopharmaceutical performance.

## 4 Comparative Analysis of IL Performance across Administration Routes

Ionic liquids exhibit route-dependent physicochemical and biological behaviors, reflecting differences in the solvent environment, tissue permeability, and formulation architecture. Integrating data from Sections 3.1–3.6, this section compares how ILs function in oral, injectable, topical, intranasal, ocular, and advanced drug-release systems, emphasizing solubility, stability, permeability, toxicity, and manufacturability.

### 4.1 Solubility and Maintenance of Supersaturation

Across all routes, ionic pairing and hydrogen-bonding networks are central to the remarkable solubilizing capacity of the ILs. In oral formulations, hydrophilic and amphiphilic ILs increase dissolution rates by two to 4 orders of magnitude, preventing recrystallization and enhancing bioavailability through microenvironmental pH modulation.^29–36^ Injectable systems depend on polar, biocompatible ILs that maintain monomeric drug states—such as amphotericin B and paclitaxel—while replacing toxic surfactants like Cremophor EL.^7,50–53^ In topical and transdermal formulations, ILs combine solubilization with interfacial structuring, forming gels, micelles, and nanoemulsions that sustain local drug reservoirs.^57–66^ Nasal and ocular preparations exploit similar ionic-solvation effects within mucoadhesive matrices to increase residence time and absorption.^97–105^ Advanced IL platforms, including hydrogels, ionogels, and polymeric implants, extend this principle to solid or semisolid systems that couple solubility control with sustained or stimuli-responsive release.^106–109^

### 4.2 Chemical and Colloidal Stability

The stabilization mechanisms of ILs vary with the formulation environment. Oral ILs suppress polymorphic transitions and degradation during digestion.^29–31^ Injectable formulations demand long-term colloidal and chemical stability under physiological ionic strength and temperature.^7,12,50–53^ Topical and intranasal ILs stabilize emulsions and soft matrices through ionic self-assembly, maintaining phase integrity during storage and application.^11,67–75,98,99^ Ocular ILs preserve labile drugs such as diacerein or pilocarpine by maintaining physiological pH and limiting hydrolysis.^104,105^ Stimuli-responsive and redox-active ILs used in advanced delivery systems introduce an additional level of control, allowing drug release or activation under acidic, oxidative, or thermal conditions.^106–109^

### 4.3 Permeability and Transport Mechanisms

IL-induced permeability arises from the reversible reorganization of biological barriers. In the gastrointestinal tract, ILs promote transcellular diffusion and transient opening of tight junctions.^10,31,41–44^ In parenteral systems, IL coatings on nanoparticles reduce protein adsorption and prolong circulation time while preserving hemocompatibility.^54–56^ In dermal applications, ILs fluidize stratum-corneum lipids, increase diffusion coefficients, and enable controlled delivery without irritation.^62–72^ Within the nasal mucosa, ILs reduce mucus viscosity and transiently modulate junctional proteins to achieve N2B transport.^97–100,102,103^ In ocular systems, species that remain largely un-ionized at tear pH enhance corneal flux and retention.^104,105^ Advanced IL-based hydrogels, microneedles, and ionogels further exploit these effects to achieve controlled, on-demand transport governed by magnetic, thermal, or chemical triggers.^107,108^

### 4.4 Toxicity and Biocompatibility

Toxicological profiles depend strongly on the ion structure and physicochemical parameters. Long-chain imidazolium cations and fluorinated anions often induce membrane disruption and oxidative stress, restricting clinical use.^59,88,119^ Consistent with broader toxicological studies, long-chain imidazolium and pyridinium salts have been shown to perturb membranes and promote mitochondrial stress in mammalian and microbial models, whereas cholinium- and amino-acid–based ILs display markedly lower cytotoxicity and improved biodegradability.^121–123^ Across pharmaceutical applications, choline-derived ILs produce reversible barrier effects and favorable biocompatibility profiles in oral, topical, intranasal, and ocular systems.^10,30,31,41–44,46–48,102,104,105^

For parenteral use, hemolysis, complement activation, and osmolality remain decisive safety parameters,^7,50–56^ while covalently bound or polymer-integrated ILs in advanced materials minimize leaching and enhance long-term compatibility.^110–116,120^ High-content imaging and in silico quantitative-structure–activity models have identified molecular descriptors governing IL toxicity, supporting rational structure–toxicity design.^124,125^ In parallel, OECD-aligned ecotoxicity frameworks and pharmacokinetic modeling of API–ILs illustrate the growing regulatory maturity of case-by-case safety assessment.^126–128^ Collectively, these findings indicate that judicious ion-pair selection can reconcile the formulation functionality with acceptable toxicological and environmental profiles.

### 4.5 Manufacturability and Dosage-Form Design

Oral ILs are frequently transformed into solid dispersions via spray-drying or adsorption on mesoporous carriers.^45–48^ Injectable formulations must comply with rigorous limits on residual solvents and ionic strength while maintaining isotonicity.^7,12,50–53^ Topical and nasal ILs exploit self-assembled gels and emulsions with tunable viscosity,^11,67–76,101–103^ whereas ocular systems require optical clarity and osmolar balance for comfort.^104,105^ In advanced release technologies, ILs serve as structural building blocks within hydrogels and implants, enabling sustained release from hours to months using scalable polymer-processing methods.^114–116,120^

### 4.6 Functional Convergence and Mechanistic Diversity

Despite the diversity of routes, common molecular principles underpin IL performance: ionic solvation and hydrogen bonding drive enhanced dissolution;^29–31,50,51^ dynamic interfacial reorganization governs permeability;^10,41–44,54–56^ and charge-mediated assembly stabilizes colloidal structures.^69–73,110–113^ The expression of these mechanisms is route-specific—oral systems emphasize dissolution, injectable systems emphasize safety and isotonicity, topical and nasal systems rely on reversible barrier modulation, ocular systems prioritize mildness and transparency, and advanced IL platforms integrate multiple functionalities into unified, programmable materials.

### 4.7 Outlook and Translational Considerations

Cross-route comparisons reveal both opportunities and challenges. ILs allow precise tuning of solubility, stability, and permeability, yet their successful translation depends on standardized evaluation of chronic toxicity, long-term safety—particularly for ocular and nasal systems—and scalable synthesis with controlled impurity levels. These aspects remain critical for advancing IL-based formulations from a preclinical proof of concept to regulatory acceptance.

## 5 Current Trends and Future Perspectives

The field of ILs has entered a transformative phase in which their role is expanding from solubilizing excipients to programmable molecular tools capable of controlling drug disposition, tissue responses, and therapeutic precision. Several converging trends are shaping this evolution.

### 5.1 Bioinspired and Degradable IL Architectures

A major trajectory in IL research involves the development of bio-orthogonal, metabolizable, and task-specific ILs derived from endogenous metabolites (e.g., choline, amino acids, and organic acids) and enzymatically cleavable anions. These designs aim to reconcile high pharmaceutical performance with predictable biodegradation profiles, reduced ecotoxicity, and improved acceptability in regulatory risk assessments. Emerging classes—such as zwitterionic ILs, hybrid deep eutectic systems, and transient ionic assemblies—illustrate how soft, biomimetic interactions can be leveraged to achieve drug loading without compromising biocompatibility.

### 5.2 Computation-Accelerated Design and Predictive Toxicology

Recent advances in AI-driven molecular generation, multiscale molecular dynamics, and quantum-informed force fields are redefining how ILs can be designed for pharmaceutical use. Machine-learning models trained on physicochemical space unique to ILs are beginning to forecast drug–IL miscibility, protein stability, membrane perturbation, and cytotoxicity with unprecedented fidelity. These tools will soon allow medicinal chemists to perform inverse design of ion pairs optimized for specific therapeutic tasks—such as dermal penetration, mucus transport, or endosomal escape—prior to synthesis. AI-guided prediction of biodegradability and off-target interactions will be crucial to accelerating regulatory acceptance.

### 5.3 Hybrid IL-Based Materials as Adaptive Therapeutic Matrices

A second trend involves hierarchical hybrid systems—including ionogels, IL–polymer composites, IL-templated nanoparticles, and IL-enabled microneedles—that integrate mechanical robustness with dynamic physicochemical behavior. These platforms function as adaptive drug depots responding to local pH, oxidative stress, ionic strength, or electromagnetic fields. As IL chemistry becomes increasingly modular, it is now possible to tune mesoscale structure (e.g., vesicular compartments, bicontinuous phases, ordered nanodomains) to orchestrate spatiotemporal release, enhance stability of labile biologics, or synergistically modulate host–microbe interactions.

### 5.4 Precision Delivery and Biological Interfacing

Future pharmaceutical ILs will expand beyond solubilization toward active biological interfacing, including tight-junction regulation, mitochondrial targeting, redox-modulated release, epigenetic modulation, and immunoadjuvant activity. IL-enabled gene therapy, protein stabilization, and vaccine formulations exemplify this shift. Next-generation ILs may function as molecular actuators, coupling reversible physicochemical changes to biologically meaningful outputs, such as enhanced mucociliary transport, antigen presentation, or controlled ferroptosis.

### 5.5 Translational Barriers and Regulatory Frameworks

Despite their promise, ILs occupy a unique regulatory space at the intersection of excipients, APIs, and novel chemical entities. The most pressing translational challenges include:

i. standardized toxicological protocols that account for IL-specific properties (ionicity, hydrogen-bond networks, aggregation phenomena);
ii. pharmacokinetic and biodistribution models that explicitly incorporate ionic speciation and ion-pair dynamics;
iii. environmental fate pathways, including biodegradation and aquatic toxicity;
iv. GMP-compliant manufacturing, ensuring batch-to-batch consistency in IL composition, residual solvent content, and water activity.

International harmonization of evaluation frameworks—particularly through the ICH, EMA, FDA, and OECD routes—will be essential to unlock clinical translation.

### 5.6 Convergence with Sustainability and Circular Chemistry

The future of IL-enabled pharmaceuticals will increasingly intersect with green chemistry and circular-materials design. Emerging biodegradable ILs, renewable feedstock synthesis, low-energy ion-pair assembly, and IL recovery and recycling strategies offer new pathways for sustainable drug manufacturing. These developments are particularly relevant as pharmaceutical companies commit to reducing their carbon footprint and minimizing persistent organic pollutants.

### 5.7 Toward Clinically Relevant IL-Enabled Technologies

Successful translation will likely emerge first in high-value therapeutic niches where ILs provide clear advantages: (i) Stabilization of fragile biologics and vaccines; (ii) Transdermal and mucosal delivery of macromolecules; (iii) Long-acting depots for chronic diseases; (iv) Infection-responsive or inflammation-triggered release systems; (v) Biocompatible microneedles and ocular depots; and (vi) IL-enabled antivirals, anticancer systems, and gene therapeutics.

Strategic collaborations between academia, industry, and regulatory agencies—coupled with advances in IL cheminformatics, materials science, and pharmacology—will be critical to move IL formulations from experimental concepts to next-generation, sustainable pharmaceutical platforms.

Collectively, these trends illustrate a paradigm shift: ILs are evolving from passive solvents to engineered molecular technologies capable of modulating biological systems with precision. The next decade will determine whether ILs can fulfill their promise as clinically viable, environmentally responsible, and functionally transformative agents in drug delivery.

## 6 Conclusions

This review consolidates current knowledge on the structure–function–bioactivity relationships governing IL-based pharmaceutical formulations. Evidence across oral, injectable, topical, ocular, and nasal routes confirms that the IL chemical architecture decisively influences drug solubility, permeability, and biocompatibility. The ability of ILs to function as both solvents and active formulation components provides a powerful design platform for addressing solubility, permeability, and bioavailability challenges in contemporary pharmaceutics.

Achieving their full translational potential, however, requires the integration of rational molecular design principles with validated toxicological data and scalable manufacturing approaches. By bridging chemistry, formulation science, and toxicology, ILs represent a promising foundation for the next generation of adaptive, safe, and multifunctional drug-delivery systems capable of meeting emerging therapeutic and regulatory demands.

Key Take-Home Insights

1. **IL structure dictates function:** Small structural changes (cation/anion identity, H-bond donors/acceptors, hydrophobicity) drastically influence solubility, permeability, and toxicity.
2. **API–ILs overcome solubility and polymorphism limitations:** Converting poorly soluble or unstable drugs into IL forms enables predictable dissolution and eliminates undesired crystallization.
3. **ILs modulate biological barriers:** ILs can transiently modulate mucus, epithelium, and tight junctions, enabling enhanced oral, nasal, ocular, and transdermal absorption.
4. **Biocompatible and bioinspired ILs are emerging:** Choline, amino-acid based, and biodegradable ILs address sustainability and regulatory expectations.
5. **Ionogels, polymer–IL hybrids, and IL-templated nanocarriers create adaptive matrices:** These systems tune mechanical strength, release kinetics, and responsiveness to pH, temperature, magnetic fields, and light.
6. **ILs can act as active pharmacological agents:** Some ILs possess intrinsic antimicrobial, anti-inflammatory, antioxidant, or membrane-active properties, enabling dual-function formulations.
7. **ILs unlock challenging administration routes:** Nose-to-brain, ocular, and transdermal delivery platforms benefit from IL-mediated solubilization and reversible barrier modulation.
8. **ILs stabilize sensitive biologics:** Certain choline and fluorinated ILs preserve protein conformation, vaccine antigens, and nucleic acids, expanding IL use into biopharmaceuticals and gene therapy.
9. **Predictive modeling and AI accelerate IL formulation design:** Computational tools are beginning to predict IL–drug compatibility, toxicity, and permeability, reducing empirical screening.
10. **Regulatory frameworks remain the key bottleneck:** Standardized toxicology, biodegradability testing, and manufacturing guidelines are urgently needed for clinical translation.
